# Genome-wide transcriptome analysis reveal the molecular mechanism for triggering the formation of purple leaves in rice mutants *nip-lpl* and *nip-dpl*

**DOI:** 10.3389/fpls.2025.1584423

**Published:** 2025-05-30

**Authors:** Chengyu Wang, Hongyu Zhao, Yujie Zhou, Haipeng Zhang, Xinyu Chen, Weifang Liang, Han Zheng, Fan Hou, Junjie Zhang, Liujie Xie, Mingwei Zhao, Bingsong Zheng, Jianzhong Li

**Affiliations:** ^1^ College of Agronomy, Anhui Agricultural University, Hefei, China; ^2^ Zhuji Agricultural Technology Extension Center, Zhuji, China; ^3^ College of Plant Protection, Shenyang Agricultural University, Shenyang, China; ^4^ College of Plant Protection, Yunnan Agricultural University, Kunming, China; ^5^ Wuwangnong Seed shareholding Co., Ltd., Hangzhou, China; ^6^ Guangzhou Gene Denovo Biotechnology Co., Ltd, Guanzhou, China; ^7^ Taizhou Academy of Agricultural Sciences, Taizhou, China; ^8^ National Key Laboratory for Development and Utilization of Forest Food Resources, Zhejiang A&F University, Hangzhou, China; ^9^ Qujiang District Agricultural Technology Extension Center, Quzhou, China

**Keywords:** transcriptome, WGCNA, purple leaf mutation, anthocyanin, secondary metabolism

## Abstract

**Introduction:**

The color of rice leaves are important agronomic traits that directly influence the proportion of sunlight energy utilization and ultimately affect the yield and quality, so it is crucial to excavate the mechanism of regulating rice leave color.

**Methods:**

To investigate the molecular mechanism that triggers the purple color in rice leaf, phenotypic characterization and genome-wide transcriptome analysis were conducted using the japonica rice cultivar nipponbare (Nip) and its two purple leaf mutants, *nip-light purple leaf (nip-lpl)* and *nip-deep purple leaf (nip-dpl)*, are rice purple leaf mutants from Nip’s EMS mutant library.

**Results:**

A total of 2247, 5484, 4525, 2103, 4375 and7029DEGs (differentially expressed genes) were identified in nip-a vs *nip-lpl-a*, nip-a vs *nip-dpl-a*, nip-c vs *nip-dpl-c*, nip-c vs *nip-lpl-c*, *nip-lpl-c* vs *nip-dpl-c*, *nip-lpl-a* vs *nip-dpl-a*, respectively. KEGG (Kyoto Encyclopedia of Genes and Genomes) analysis showed that the DEGs were significantly enriched in phenylalanine biosynthesis, terpenoid backbone biosynthesis, secondary metabolite biosynthesis, and hormones. Additionally, WGCNA (Weighted Gene Co-expression Network Analysis) showed that the darkmagenta module was associated with the purple color mainly due to the accumulation of anthocyanin in the leaves of the mutant rice. This module revealed three pathways for anthocyanin synthesis: phenylalanine could be catalyzed by phenylalanine lyase and cinnamic acid hydroxylase, etc., to generate dihydroxyflavone and ultimately anthocyanin. Furthermore, we speculated that the elevated expression of three hub genes (*PAL*, *CHI* and *CHS*) in *nip-lpl/dpl* leads to increased anthocyanin content relative to Nip.

**Discussion:**

These results not only revealed the molecular mechanism triggering leaf purple color in the rice mutants *nip-lpl/dpl* but also contributed greatly to identified potential genetic engineering targets for breeding anthocyanin-rich rice.

## Introduction

Rice (*Oryza sativa L.*) is one of the most important food crops in the world. Half the world’s population is dependent on rice as the primary staple food and the Asian region produces and consumes over 90% of the world’s rice (Chakuton et al., 2012). In addition to common white rice varieties, there are pigmented varieties such as the colored black, red, brown, or purple ones. In recent years, various rice mutants including rice leaf color mutation have emerged (Li et al., 2022b). Studies have shown that rice leaf color mutation is regulated by photosynthesis, gene regulation, pigment accumulation and other pathways, but the main reason is caused by the accumulation of pigment in leaves. There are three main pigments affecting leaf color mutation: chlorophyII, carotenoid and anthocyanin, among which anthocyanin is the main cause of red and purple leaf color mutation (Zhao et al., 2020). Anthocyanin is a natural water-soluble pigment in nature, which is ubiquitous in plants and influence the color of plant leaves, petals, fruits or seed coats, showing blue, red or purple according to different accumulation levels. Anthocyanins can be accumulated in rice leaves, panicles, seed coats and other parts, so that rice contains different color mutations. However, the mechanism of anthocyanin regulating rice leaf color remained still unclear (Wu et al., 2020; Yang Y et al., 2020).

Anthocyanin is one of the important secondary metabolites and it is also an antioxidant in plants (Alice et al., 2018). By measuring the photosynthetic indexes of different varieties of rice, it was found that the activity of antioxidant enzymes in rice materials containing anthocyanins was much higher than that of ordinary varieties (Chen T et al., 2022). In addition, it was found that light could induce the accumulation of anthocyanin and the transcription of anthocyanin-related genes in rice, including *DFR* (dihydroflavonol 4-reductase), *ANS* (anthocyanidin synthase), *CHI* (chalcone isomerase) and *PAL* (Phenylalanine ammonia-lyase). Besides, the UV-B and phytochrome can also promote the synthesis of *PAL (* Araguirang and Richter, 2022; Zirngibl ME et al., 2022). The primary anthocyanidins responsible for the wide range of plant colors are cyanidin, delphinidin, pelargonidin, peonidin, malvidin, and petunidin (Yamuangmorn and Prom-U-Thai, [[NoYear]]; Zhang J and Liu, 2022). In rice, the types of anthocyanins are mainly cyanidin and peonidin methylated from cyanidin. For example, the flag leaf, rice husk and glume peel of purple rice variety *purpleputtu* contain anthocyanins, the main component is cyanidin, the secondary component is paeoniflorin, and the anthocyanin content is the highest in the glume peel, followed by rice husk and flag leaf (Reddy et al., 1995). Anthocyanin belongs to flavonoids, and its synthetic precursor is phenylalanine (Zheng et al., 2019; Nuraini et al., 2020; Günther CS et al., 2022). Phenylalanine is catalyzed by phenylalanine lyase (PAL), cinnamic acid hydroxylase (C4H) and coumaric acid CoA ligase (4CL) to form the primary substrate 4-coumaroyl-CoA(4CoA). Subsequently, under the action of chalcone synthase (CHS), 4-coumaroyl-CoA and malonyl-CoA derived from fatty acid metabolism synthesize yellow chalcone in a ratio of 1:3. The chalcone is catalyzed by chalcone isomerase (CHI) and flavanone 3-hydroxylase (F3H) to synthesize the common precursor of flavonoids, dihydroflavonol (Meng L and Wang, 2021; Qiao W, 2021; Dai et al., 2022; Fu et al., 2022). Dihydroflavonol is converted into different kinds of anthocyanins in two ways. It can be directly used as a substrate for dihydroflavonol 4-reductase (DFR) and anthocyanin synthase (ANS) to synthesize colored anthocyanins (pelargonidin). It can also be hydroxylated by flavonoid 3’-hydroxylase (F3’H) or flavonoid 3’-5’-hydroxylase (F3’5’H), and then it was catalyzed by DFR and ANS to synthesize other anthocyanins (cyanidin or delphinidin) (Song X et al., 2022; Xu Z et al., 2022). Finally, through the catalysis of glucosyltransferase, anthocyanins form glycosidic bonds with one or more different carbohydrates, and finally form stable anthocyanins, which can be glucose, rhamnose, galactose, etc (Sun et al., 2019). In a previous study, *OsC1*, *OsRb* and *OsDFR* were identified as the determinants of anthocyanin biosynthesis in rice leaves (Zheng et al., 2019). Differentially expressed genes and metabolites were found in the *indica* and *japonica* backgrounds, indicating that OrC1 activated the anthocyanin biosynthetic genes *OsCHI*, *OsF3H* and *OsANS* and produced six metabolites independently (Qiao et al., 2021). To date, no studies have reported the anthocyanin accumulation mechanisms in the rice with different color mutations. Comprehending the varied genes compositions of rice colors is crucial for evaluating their introduction, and growth of superior rice cultivar.

In the previous study, we constructed a rice mutant library by mutagenesis of japonica rice (*Oryza sativa*) cultivar nipponbare (Nip) with ethyl methane sulfonate (EMS). Among the mutants produced, a rice mutant, designated nipponbare-light purple leaf (*nip-lpl*), displayed the light purple color in its flag leaves compared to its wide type rice Nip, and another one, designated nipponbare-deep purple leaf (*nip-dpl*), showed the deep purple color in its third leaves, which both provided important materials for studying the formation mechanism of purple rice leaves. In the present research, we performed phenotypic characterization and genome-wide transcriptome sequencing on two rice mutants and the wild-type Nip. As a result, we revealed three DEGs (*PAL*, *CHS* and *CHI*) of the anthocyanin biosynthesis pathway in rice as potential candidates for the regulation of anthocyanin synthesis. These findings provided significant insight into the molecular regulatory mechanism underlying anthocyanin accumulation and purple leaf formation in rice.

## Materials and methods

### Plant materials

The wild-type, Oryza sativa ssp. japonica cultivar Nip is preserved in our laboratory in Zhejiang Academy of Agricultural Sciences. Two mutants, *nip-lpl* and *nip-dpl*, are rice purple leaf mutants from Nip’s EMS mutant library; 1) The seeds were soaked in clean water for 16h, then treated with 0.5% EMS at 28°C for 4h, and then sown and collected (M1). (2) The seeds of the M1 generation were soaked in water for 16h, treated with 0.5% EMS at 28°C for 4h, and then treated with 0.7% EMS for 4h, sowing and harvesting the seeds of the M2 generation. (3) Sowing the M2 generation and screening mutant plants. Rice was sown at the nursery of Zhejiang Academy of Agricultural Sciences in Hangzhou, China, and 25-day-old seedlings were transplanted to experimental fields. In each experiment, the plots were arranged in a completely randomized block design, with a plant spacing of 20 cm in each row and a row spacing of 35 cm. In addition, field management followed normal agricultural practices. The leaves at two different positions including flag leaves and third leaves of the three rice materials were collected for transcriptome analysis. Three biological replicates were set for each sample, and 25 mg leaves were selected from each plant at the five-leaf stage. At the five-leaf stage and maturity stage, 8 plants were randomly selected for agronomic traits testing. The growth period of plants was calculated from the second day of seedling to maturity.

Plant height (cm): At two growth stages—the five-leaf stage and the maturity stage—eight rice plants were randomly selected from each plot, and the length from the ground to the apical growing point of the main stem was measured. The average value was then calculated.

1000-grain weight: Mature rice seeds from three varieties were counted to one thousand grains each, and their weight was measured using a precision electronic balance. Six replicates were set up for each variety. The thousand-grain weight was recorded to evaluate the plumpness and yield potential of rice seeds from different varieties.

Dry Matter Weight: At the maturity stage of rice, six plants were randomly selected from each treatment as biological replicates. The panicles and roots of the plants were removed, and surface soil was cleaned off. The dry matter accumulation was determined using the direct drying and weighing method, with the following specific steps: The separated plant samples were placed in an oven, first deactivated at 105°C for 30 minutes to halt physiological activity, then dried at 85°C until a constant weight was achieved. After drying, the samples were removed and cooled to room temperature in a desiccator before being weighed using a precision electronic balance to record the dry weight. Each treatment was measured in triplicate to evaluate the effects of different treatments on rice dry matter accumulation.

Number of grains per spike: Carefully cut the rice panicles and place them in a well-ventilated, dry area to dehydrate naturally for 3–5 days. Once the lemma and palea can be easily separated from the grains, proceed with measurement. Manually remove unfilled or empty grains (visually identifiable: filled grains are plump). Strip the panicle grain by grain, separating filled grains using tweezers or a vibrating sieve, with the aid of a counter. Record the number of filled grains per panicle and the total grain count (filled + unfilled grains), then calculate the seed-setting rate (filled grains/total grains × 100%).

### Sampling and RNA extraction

At the five-leaf stage of the plant, three independent repeat samples were selected from the flag and third leaves of wild-type Nip, *nip-lpl* and *nip-dpl*. Total RNA was extracted using a Trizol kit (Invitrogen, Carlsbad, CA, USA) according to the manufacturer ‘s protocol. RNA quality was assessed on an Agilent 2100 bioanalyzer (Agilent Technologies, Palo Alto, CA, USA) and examined using RNase-free agarose gel electrophoresis. mRNA was fragmented with divalent cations in NEB Fragmentation Buffer, and libraries were constructed using either the NEB standard or strand-specific method. The library was then quantified with Qubit 2.0 and diluted to 1.5 ng/µl. The insert size was evaluated using Agilent 2100. After confirming the expected insert size, RT-qPCR was used to quantify the library concentration, ensuring it exceeded 2 nM for quality assurance. Libraries were pooled based on their effective concentration and the required sequencing data volume for Illumina sequencing. DESeq2 software performed differential expression analysis on samples with biological replicates between the two groups. The Benjamini-Hochberg method was used to adjust P-values for controlling false discovery rates, identifying genes with adjusted P-values under 0.05 as differentially expressed.

### cDNA library construction and sequencing

After total RNA was extracted, eukaryotic mRNA was enriched with Oligo (dT) beads. Ribo ZeroTM magnetic kit (Epicentre, Madison, WI, USA) was used to remove rRNA-enriched prokaryotic mRNA. Then, the enriched mRNA was fragmented into short fragments using fragment buffer and reverse transcribed into cDNA using Illumina ‘s NEBNext Ultra RNA library preparation kit (NEB # 7530, New England Biolabs, Ipswich, MA, USA). The purified double-stranded cDNA fragment was repaired by repairing the end, adding A base and connecting to the Illumina sequencing adapter. The ligation reaction was purified with AMPure XP beads (1.0X). The size of the connected fragments was selected by agarose gel electrophoresis and polymerase chain reaction amplification. Gene Denovo Biotechnology Co. (Guangzhou, China) used Illumina Novaseq6000 to sequence the cDNA library.

### RNA sequence data screening

The readings obtained from the sequencer include raw readings containing adapters or low-quality bases, which affected subsequent assembly and analysis. Therefore, in order to obtain a high quality clean read, the reads was further filtered by fastp(version 0.18.0) (Chen et al., 2018). The parameters were as follows: (1) Remove the read containing the adapter; (2) Remove readings containing more than 10% of unknown nucleotides (N); (3) Remove the foundation containing more than 50% low quality (Q value ≤ 20). (4) Remove data containing Poly A bases.

We also compared the data with ribosomal RNA (rRNA) and reference genomes. The read sequence was mapped to the rRNA database using the short reading sequence alignment tool Bowtie2 (version 2.2.8) (Langmead and Salzberg, 2012). Then the rRNA mapped readings were removed. The remaining clean readings were further used for assembly and gene abundance calculations. At the same time, the index of the reference genome was established, and HISAT2 (Kim et al., 2015) was used to locate the paired end clean readings to the reference genome 2.4 and other parameters were set to default values.

Differentially expressed genes between two different comparison groups were analyzed by DESeq2 (Love et al., 2014) software. The absolute value of false discovery rate (FDR) <0.05 and | log2FC |>1 was used as the threshold of DEG classification. First, we mapped the differential genes to each term of the GO database (http://www.geneontology.org/) and calculated the number of differential genes for each term to obtain a list of differential genes with a certain GO function and the number of differential genes. Then, the hypergeometric test was used to find out the GO entries that were significantly enriched in the differential genes compared with the background. Furthermore, DEGs were mapped in the KEGG pathway database to identify co-enriched pathway information. The most significantly enriched pathways for DEG were identified based on DEG enrichment pathways, and a histogram was generated using GraphPad 8.0.2.

### Co-expression network analysis of module construction

In the WGCNA (v1.47) package in R language, the whole process gene set was screened. After retaining the expression level: number of transcript fragments per thousand bases/millions of mapped reads (FPKM) ≥ 1 as the threshold to screen out about 35.5% of the genes, the power value was 8, the minimum number of genes in the module was 50, and the maximum number of modules was 20. The TOM value retained the top 1000 relationship pairs and clustered into 17 related modules. In order to determine the relationship between modules and specific leaf position expression in wild type and mutant, the correlation coefficient was calculated as the module eigenvalue with the sample. The intra-module connectivity and module correlation of each gene were calculated by R package (Damian et al., 2015; Shannon and Cytoscape, 2003).

### Quantitative real-time PCR

To ensure the reliability of the RNA-Seq results, we selected a subset of differentially expressed genes and conducted qRT-PCR validation on the LightCycler 480 system (Roche, Basel, Switzerland). **Supplementary Table S1** provides details of the primers used in this study. The reaction mixture comprised a final volume of 10-µL, containing 0.2 µl of cDNA, 0.2 µl of primers, 5 µL of 2× Ultra SYBR mix, and 4.4-µL of RNase-free water. The PCR program was set as follows: an initial denaturation at 94°C for 5 minutes, followed by 30 cycles of denaturation at 94°C for 10 seconds, annealing at 60°C for 10 seconds, and extension at 72°C for 20 seconds. We utilized three biological replicates, with each replicate performed in triplicate for technical repeats. The relative expression levels (fold changes) were calculated using the 2^-ΔΔCt^ method. The significance level was set at a p-value < 0.05. All qRT-PCR assays were repeated three times to ensure the reliability of the results.

### Data processing and statistical analysis

Based on the expression information, we used R (http://www.r-project.org/) to carry out Principal Component Analysis (PCA). Reads count data obtained from gene expression level analysis was analyzed using DESeq2 software. STRING protein interaction database (http://string-db.org) was used to analyze the differential gene protein interaction network. The data were expressed as the mean ± standard deviation (SD) of triplicates. The differences between the means were analyzed using ANOVA analyses, and a p value < 0.05 was considered to be significant.

## Results

### Phenotypic characteristics of different rice varieties

**Figure 1:**
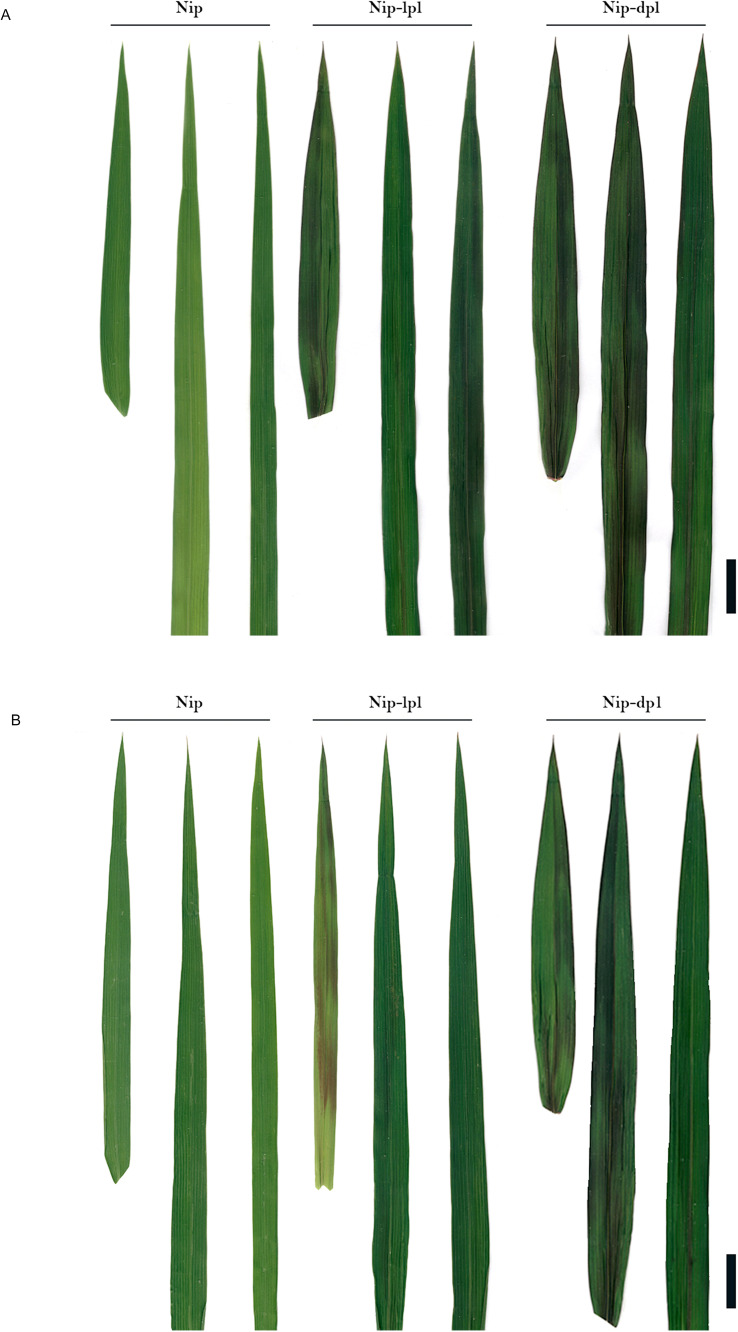
Comparison of leaf phenotypes among wild-type Nip and mutant *nip-lpl* and *nip-dpl*. **(A)** Adult-plant stage: flag leaf; top second leaf; top third leaf; **(B)** Tillering stage: flag leaf; top second leaf; top third leaf.

**Figure 2:**
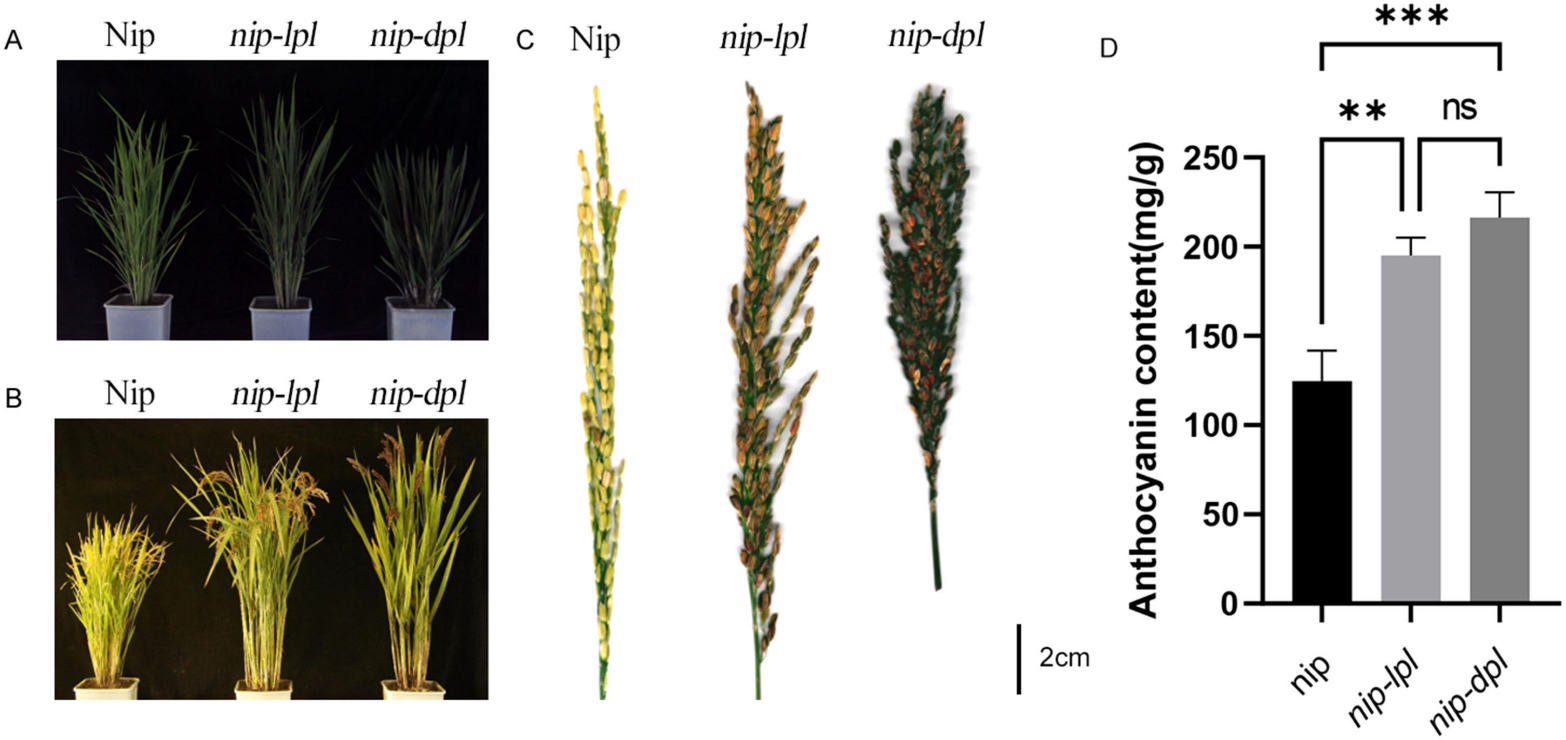
Phenotype comparison of wild-type Nip and mutants *nip-lpl* and *nip-dpl*. **(A)** Comparison of plants at tillering stage: left, Nip, middle, *nip-lpl*, right, *nip-dpl*, scale 5cm; **(B)** Comparison of mature plants: left, Nip, middle, *nip-lpl*, right, *nip-dpl*, 15cm scale; **(C)** Comparison of mature spikes: left, Nip, middle, *nip-lpl*, right, *nip-dpl*; **(D)** Quantification of total anthocyanin content in Nip, *nip-lpl*, *nip-dpl.*. ** represents p<0.01, *** represents p<0.001, ns represents the difference is not significant.

Compared with the normal wild-type nipponbare (Nip), the two purple leaf mutants, designated nip-light purple leaf (*nip-lpl*) and nip-deep purple leaf (*nip-dpl*) had leaf color mutations from the five-leaf stage to the mature stage. The leaves of *nip-dpl* appeared purple, *nip-lpl* appeared light purple, and the leaf color of *nip-dpl* was deeper than that of *nip-lpl*. In addition, the degree of leaf color mutation at different leaf positions was different in different periods. In the five-leaf stage, the flag leaf of *nip-lpl* showed various light purple mutations, the second leaves were less numerous, and the third leaf showed normal green. On the contrary, *nip-dpl* showed normal green flag leaves with fewer purple mutations in the second leaf and more purple mutations in the third leaf. Leaf color mutation from the beginning of the veins, gradually to the blade on both sides (**Figure 1A**). Especially during tillering stage, these two leaves showed the most color changes. A large area of purple mutation also appeared in the leaves at three different locations (**Figure 1B**). Both leaf color mutations start from the veins, spread to other parts of the blade. At this time, almost all the leaves of the whole plant showed leaf color mutation (**Figure 2A**). The leaf color mutation of maturity stage is significantly decreased compared with pentaleaf stage and tillering stage. The leaf color mutation of *nip-lpl* almost disappeared, and the leaf color mutation of *nip-dpl* only existed in a small amount between the veins (**Figures 1A, B**). Interestingly, the spike color of wild type Nip was green, the spike color of *nip-lpl* was light purple, and the spike color of *nip-dpl* was deep purple (**Figures 2B, C**). Furthermore, we performed the quantitative analysis of anthocyanin content in the leaves of three rice cultivars, the anthocyanin contents of *nip-lpl* and *nip-dpl* were significantly higher than that of Nip (**Figure 2D**, *p*<0.05).

**Figure 3:**
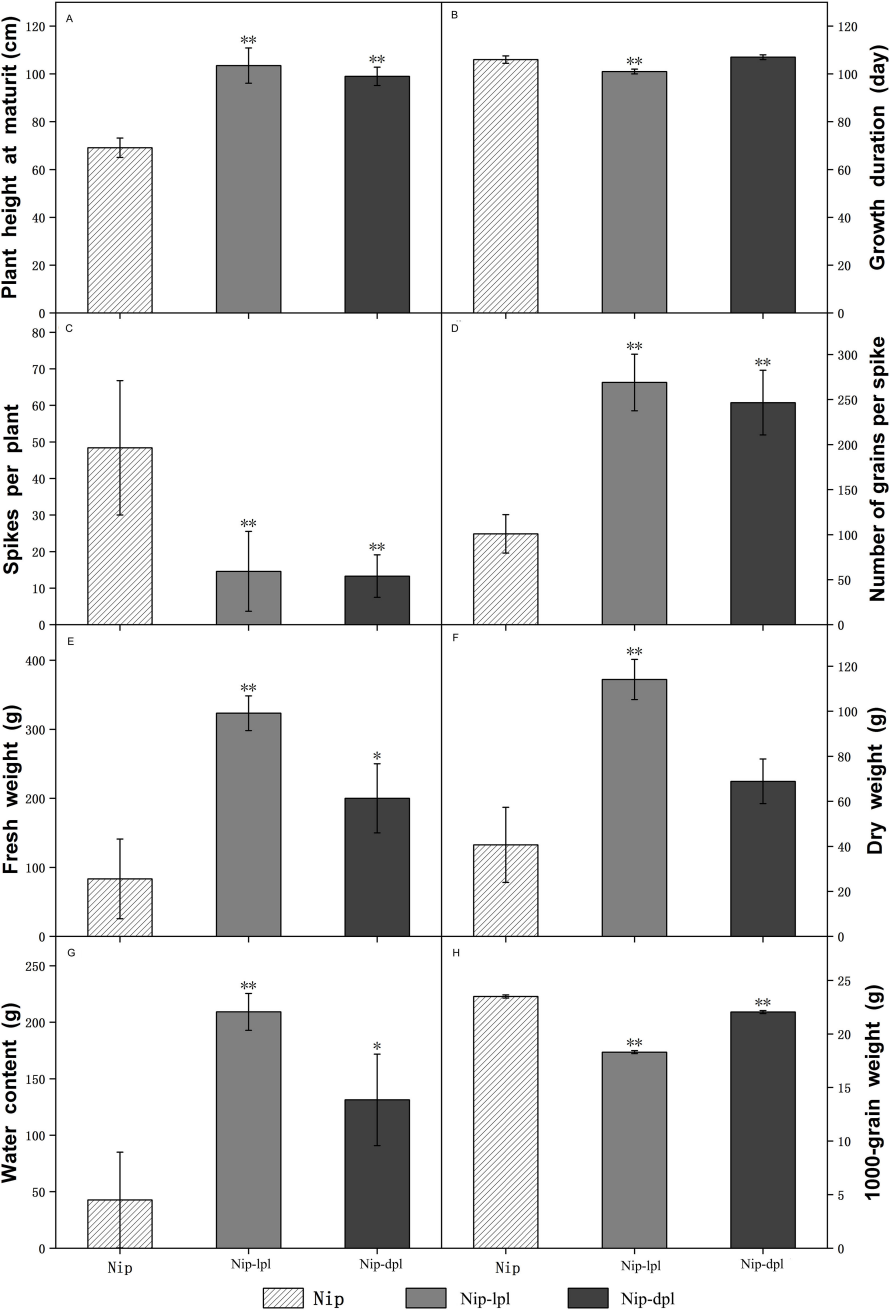
Comparison of agronomic traits between wild-type Nip and mutants nip-lpl and nip-dpl. **(A)** Plant height at maturity; **(B)** Growth duration; **(C)** Spikes per plant; **(D)** Number of grains per spike; **(E)** Fresh weight; **(F)** Dry weight; **(G)** Water content; **(H)** 1000-grain weight. The mean ± standard deviation was 8 plants. ** was significantly different when p < 0.01 determined by t-test. * was significantly different when p<0.05 determined by t-test.

In addition, we explored the different agronomic traits of the three rice materials. Among them, the growth duration and dry weight of wild-type Nip and *nip-dpl* were relatively close, and in terms of plant height, number of grains per spike, fresh weight and water content at maturity, *nip-lpl* and *nip-dpl* were much higher than wild-type Nip, but the spikes per plant and 1000-grain weight of the two mutant plants was smaller than that of wild-type Nip (**Figure 3**).

### Evaluation of RNA sequencing read and mapping results

We selected two leaves of three varieties for RNA sequencing according to the leaf phenotypes at different positions of the three rice cultivars: the flag leaf of the wild type was named nip-a, and the third leaf was named nip-c. The flag leaf of the mutant *nip-lpl* was named *nip-lpl-a*, and the third leaf was named *nip-lpl-c*, while the flag leaf of the mutant *nip-dpl* was *nip-dpl-a*, and the third leaf was *nip-dpl-c*. The minimum R value of the comparison between the three biological replicates of each sample was 91.3%, most of which were between 95.2% and 99.8% (**Supplementary Figure S1A**). Based on the expression information, we performed principal component analysis (PCA), to illustrate the repeatability between samples, and assist in excluding outliers. PC1 and PC2 explained 81.1% and 9.1% of the total variance among the samples, respectively. Three repeats of the same cultivar were clustered together and obviously separated from other cultivars. The results showed that the expression between duplicate samples was closely related (**Supplementary Figure S1B**), which was enough to prove the repeatability of samples.

### Differentially expressed gene analysis

**Figure 4:**
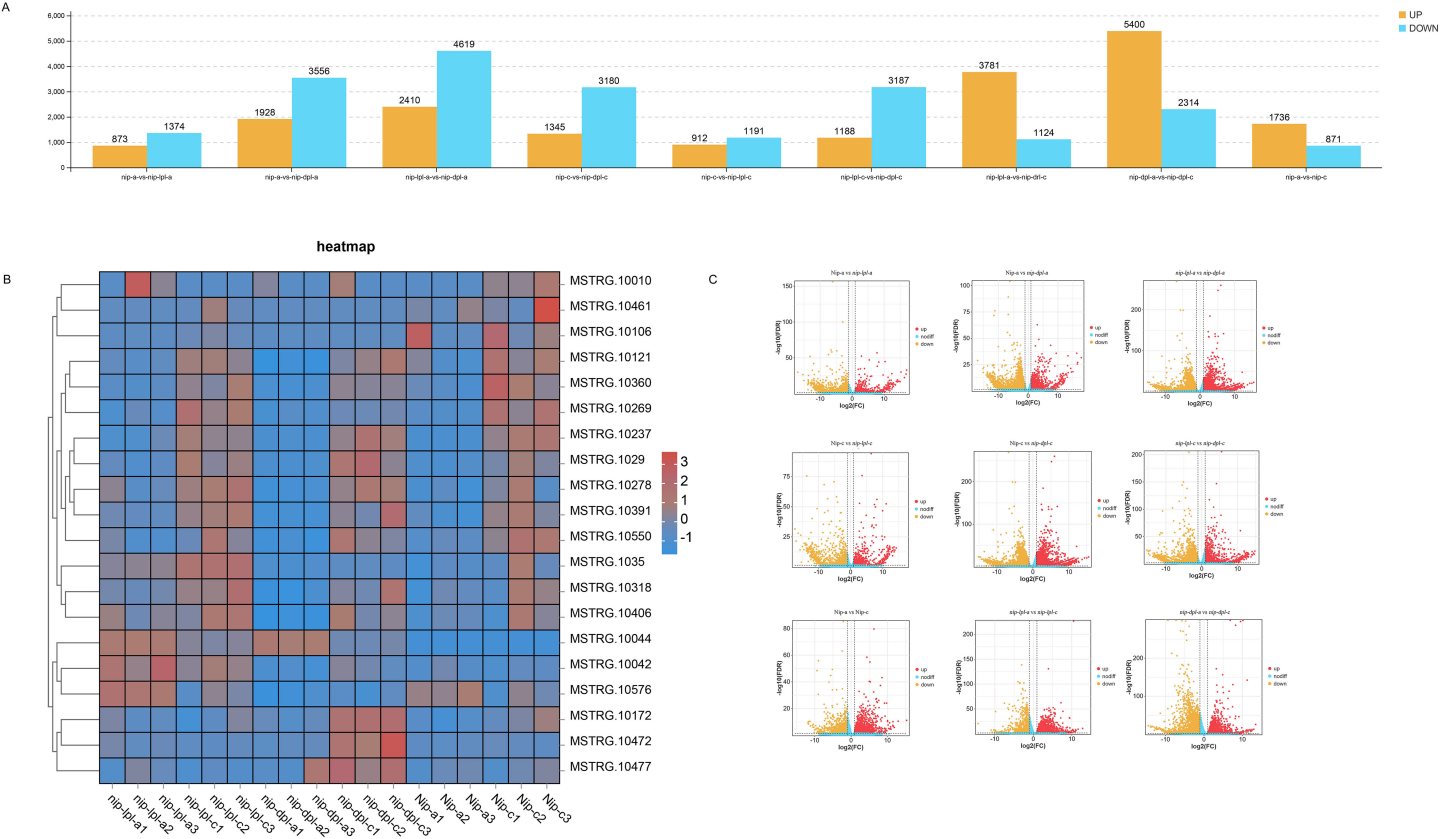
Analysis of differential gene expression in all pairwise comparisons. **(A)** The number of up-regulated and down-regulated differentially expressed genes (DEGs); **(B)** Cluster heat maps of all DEGs in the three cultivars, with relative levels of genes ranging from low (blue) to high (red). **(C)** Gene expression level in volcano plot.

A total of 2247, 5484, 4525, 2103, 4375, 7029, 4905, 7714 and 2607 DEGs were identified in nip-a vs *nip-lpl-a*, nip-a vs *nip-dpl-a*, nip-c vs *nip-dpl-c*, nip-c vs *nip-lpl-c*, *nip-lpl-c* vs *nip-dpl-c*, *nip-lpl-a* vs *nip-dpl-a*, *nip-lpl-a* vs *nip-lpl-c*, *nip-dpl-a* vs *nip-dpl-c*, and nip-a vs nip-c, respectively (**Supplementary Table S2**). As shown in **Figure 4A**, the largest number of DEGs was found in *nip-dpl-a* vs *nip-dpl-c* (7714 DEGs including 5400 up-regulated and 2314 down-regulated genes), followed by *nip-lpl-a* vs *nip-dpl-a* (7029 DEGs including 2410 up-regulated and 4619 down-regulated genes), and nip-a vs *nip-dpl-a* (5484 DEGs including 1928 up-regulated and 3556 down-regulated genes). There were 4905 DEGs in *nip-lpl-a* vs *nip-lpl-c*, of which 3781 genes were significantly up-regulated and 1124 genes were significantly down-regulated. **Figure 4B** illustrated a hierarchical heatmap clustering analysis based on DEGs concentration data, revealing three distinct clusters with varying gene expression levels in Nip, *Nip-lpl* and *Nip-dpl*. The volcano map can visually display these DEGs and their distribution patterns of up-regulated genes and down-regulated genes. It indicated that there was a significant overall gene expression pattern between different comparisons (**Figure 4C**). For example, the distribution pattern of up-regulated genes in the leaf control group at different positions of *nip-lpl* vs *nip-dpl* was much higher than that in other groups. The number of DEGs in the *nip-dpl-a* vs *nip-dpl-c* was the largest, indicating that there was a difference in expression between leaves with or without anthocyanin and anthocyanin accumulation.

### Gene ontology analysis of DEGs

**Figure 5:**
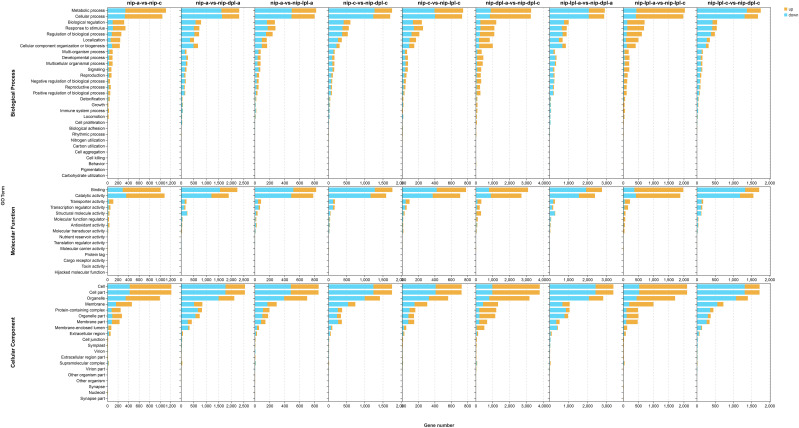
Distribution and quantity of DEG in three ontology categories, molecular functions, cell components and biological processes. The horizontal coordinate indicates the number of differential genes enriched in the items, and the vertical coordinate indicates the name of the GO items. The orange represented up-regulated DEGs, the blue represented the down-regulated DEGs.

In order to better explore the function of DEGs, we performed gene ontology analysis. In the results of GO analysis, we found that 58 GO terms were significantly enriched in the nip-a vs nip-c, which included 27 items of biological process, 11 items of molecular function, and 20 items of cellular component. DEGs in the biological process group were mostly divided into “metabolic process” (GO:0008152) and “cellular process” (GO:0009987). In the molecular function group, “catalytic activity” (GO:0003824) and “binding” (GO: 0005488) had the highest enrichment, while in the cell component group. The most abundant GO terms were “cell part” (GO:0044464), “Cell” (GO:0005623), “organelle” (GO:0043226), “membrane”(GO: 0016020) (**Figure 5**, **Supplementary Table S3**). In the comparison groups of *nip-lpl-a* vs *nip-lpl-c*, *nip-dpl-a* vs *nip-dpl-c*, the distribution patterns of the number and type of GO terms were similar to those of nip-a vs nip-c, but the number of enriched DEGs was much higher than that of nip-a vs nip-c. In the comparison group nip-a vs *nip-lpl-a*, DEGs were divided into 56 GO terms, including 25 biological processes, 13 molecular functions, and 18 cellular components. In the comparison group of nip-a vs *nip-dpl-a*, 26 GO terms were enriched in biological processes, 12 GO terms were enriched in molecular functions, and 20 GO terms were enriched in cellular components. In addition, 61 significantly enriched GO terms were identified in the *nip-lpl-a* vs *nip-dpl-a*, including 28 biological processes, 13 molecular functions, and 20 cellular components. In the comparison of nip-c vs *nip-lpl-c*, 25 GO terms were enriched in biological processes, 12 GO terms were enriched in molecular functions, and 19 GO terms were enriched in cellular components. There were 57 GO terms in the nip-c vs *nip-dpl-c*, including 26 biological processes, 11 molecular functions, and 20 cellular components. The enrichment classification of GO in the *nip-lpl-c* vs *nip-dpl-c* was very similar to the above. In these GO terms, the number of differentially down-regulated genes was much higher than that of differentially up-regulated genes (**Figure 5** **). **Kyoto Encyclopedia of Genes and Genomes analysis of DEGs

**Figure 6:**
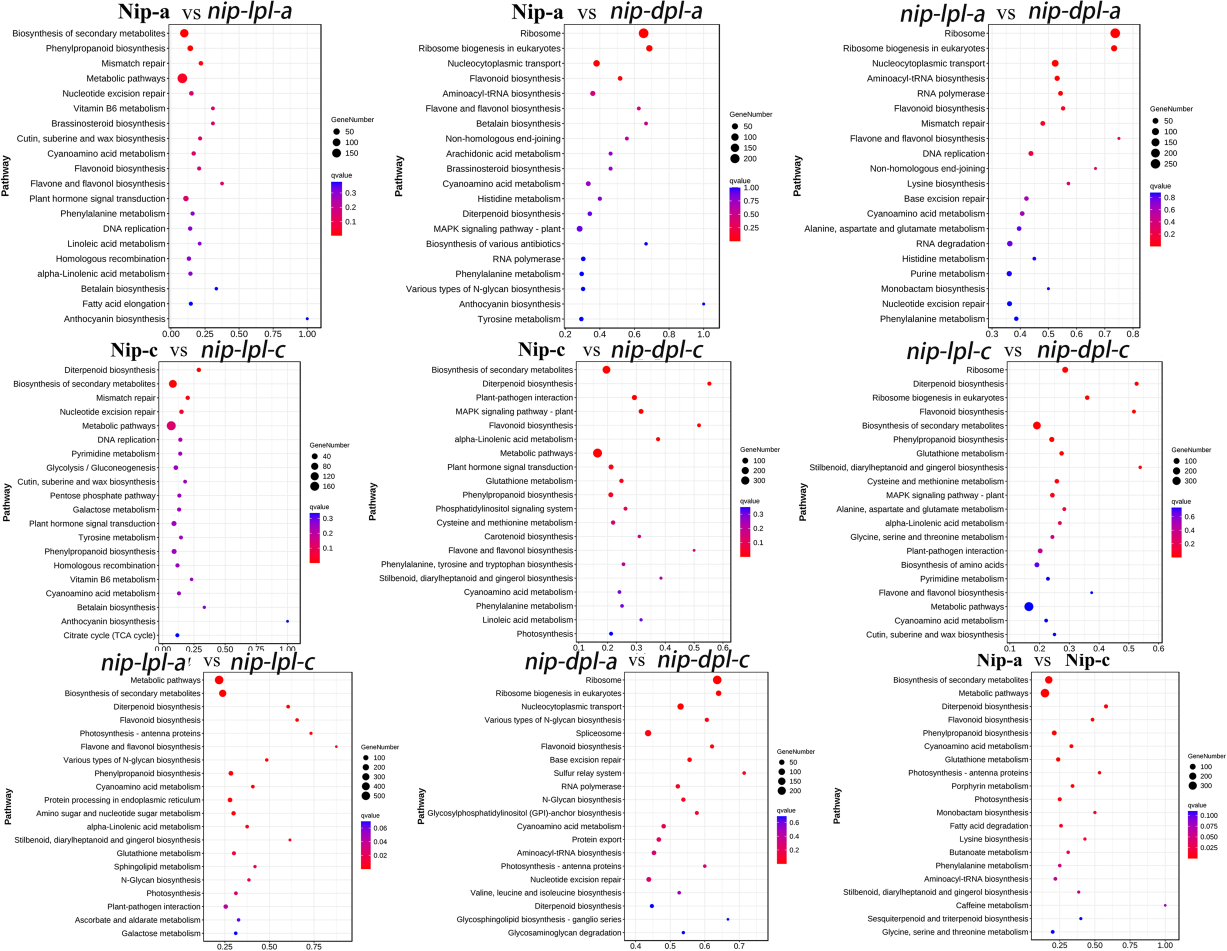
KEGG enrichment of annotated DEGs. The left Y axis represents the KEGG pathway, and the X axis represents the rich factor. The high q value is represented by blue, and the low q value is represented by red. The richness factor is the ratio of the number of DEGs mapped to a pathway to the total number of genes mapped to the pathway.

In order to further understand the biological functions of DEGs and identify the pathways involved in DEGs, we performed KEGG analysis (**Figure 6**, **Supplementary Table S4**). In the comparison groups of *nip-a* vs *nip-c*, *nip-lpl-a* vs *nip-lpl-c*, biosynthesis of secondary metabolites, metabolic pathway, flavonoid biosynthesis, diterpenoid biosynthesis had the highest enrichment degree. In the *nip-dpl-a* vs *nip-dpl-c*, ribosome, ribosome biogenesis in eukaryotes, RNA transport and spliceosome were highly enriched. The highest enrichment pathways of nip-a vs *nip-lpl-a* were biosynthesis of secondary metabolites, metabolic pathway, phenylpropanoid biosynthesis and mismatch repair. In the nip-a vs *nip-dpl-a* and *nip-lpl-a* vs *nip-dpl-a* comparison groups, ribosome, ribosome biogenesis in eukaryotes, RNA transport, flavonoid biosynthesis, aminoacyl-tRNA biosynthesis and other pathways were highly enriched. Biosynthesis of secondary metabolites and metabolic pathway were enriched in the nip-c vs *nip-lpl-c* as well as nip-c vs *nip-dpl-c*. In addition, the pathways of diterpenoid biosynthesis, plant-pathogen interaction, flavonoid biosynthesis and nucleotide excision repair were enriched in the *nip-lpl-c* vs *nip-dpl-c*.

### Identification of common DEGs in comparison groups of different rice materials

In the leaf comparison group of three rice materials at the same position, we analyzed 71 common DEGs (nip-a vs *nip-lpl-a*, nip-a vs *nip-dpl-a*, *nip-lpl-a* vs *nip-dpl-a*, nip-c vs *nip-lpl-c*, nip-c vs *nip-dpl-c*, *nip-lpl-c* vs *nip-dpl-c*) (**Supplementary Figure S2**). In addition, we also analyzed the differences between the corresponding leaves of two different rice materials. We identified 1104 common DEGs among nip-a vs *nip-lpl-a* and nip-c vs *nip-lpl-c* (**Supplementary Figure S3A**). KEGG analysis showed that these DEGs were highly expressed in biosynthesis of secondary metabolites, metabolic pathway, diterpenoid biosynthesis, phenylpropanoid biosynthesis, cyanoamino acid metabolism and mismatch repair (**Supplementary Figure S3B**). We identified 2288 common DEGs among nip-a vs *nip-dpl-a*, nip-c vs *nip-dpl-c* (**Supplementary Figure S4A**). KEGG analysis showed that these DEGs were enriched in ribosome, ribosome biogenesis in eukaryotes, flavonoid biosynthesis, phenylalanine metabolism and other pathways (**Supplementary Figure S4B**). Finally, we identified 2383 common DEGs among *nip-lpl-a* vs *nip-dpl-a*, *nip-lpl-c* vs *nip-dpl-c* and *nip-lpl* vs *nip-dpl* (**Supplementary Figure S5A**). KEGG enrichment analysis showed that these DEGs were abundantly expressed in ribosome, ribosome biogenesis in eukaryotes, RNA transport, flavonoid biosynthesis and diterpenoid biosynthesis (**Supplementary Figure S5B**).

### Structural genes involved in anthocyanin biosynthesis

**Figure 7:**
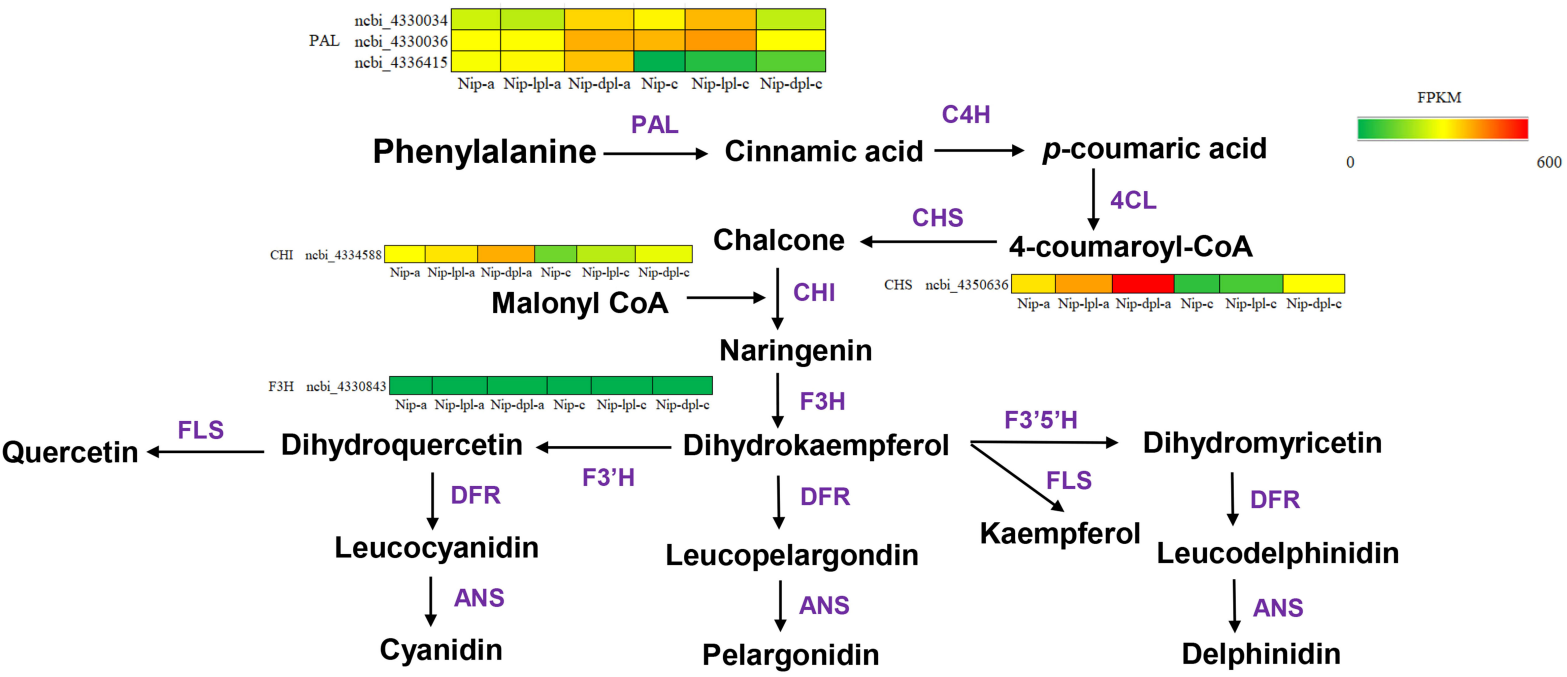
Expression levels of structural genes involved in anthocyanin biosynthesis pathway in rice. The heatmaps indicate the expression/content of respective structural genes in three rice cultivars. The percentile value of FPKM ranging from low to high is represented by green to red. The key enzymes in the anthocyanin synthesis pathway are shown below: PAL, phenylalanine ammonia lyase; C4H, cinnamate 4-hydroxylase; 4CL, 4-coumarate: CoA ligase; CHS, chalcone synthase; CHI, chalcone isomerase; F3H, flavanone 3-hydroxylase; F3’H, flavonoid 3’-hydroxylase; F3’5’H: flavonoid-3’,5’-hydroxylase; DFR, dihydroflavonol 4-reductase; ANS, anthocyanidin synthase; and UFGT, flavonoid 3-O-glucosyltransferase.

In these pathways, it was found that there were related genes involved in anthocyanin synthesis, including *CHI*, *CHS*, *DFR*, *F3H*, *ANS* and so on. In addition, other anthocyanin synthesis genes were also found in phenylalanine metabolism and phenylpropanoid biosynthesis, such as *PAL* and *4CL (* Huang et al., 2024). There were genes related to the promotion of glucose decomposition, beta-glucosidase, and antioxidant gene peroxidase. These genes were significantly up-regulated in Nip vs *nip-dpl* comparison, and partially up-regulated in other comparisons (**Supplementary Figure S6**-**Supplementary Figure S7**). Furthermore, the study analyzed hub genes in the anthocyanin biosynthesis pathway using transcriptomic data to predict the molecular mechanisms responsible for the color variations in rice (**Figure 7**). **Figure 7** illustrated that the *nip-dpl-a* exhibited elevated expression levels of *PAL*, *CHI* and *CHS* genes from the upstream synthesis pathway, followed by the *nip-lpl-a* cultivar.

### Weighted gene co-expression network analysis

**Figure 8:**
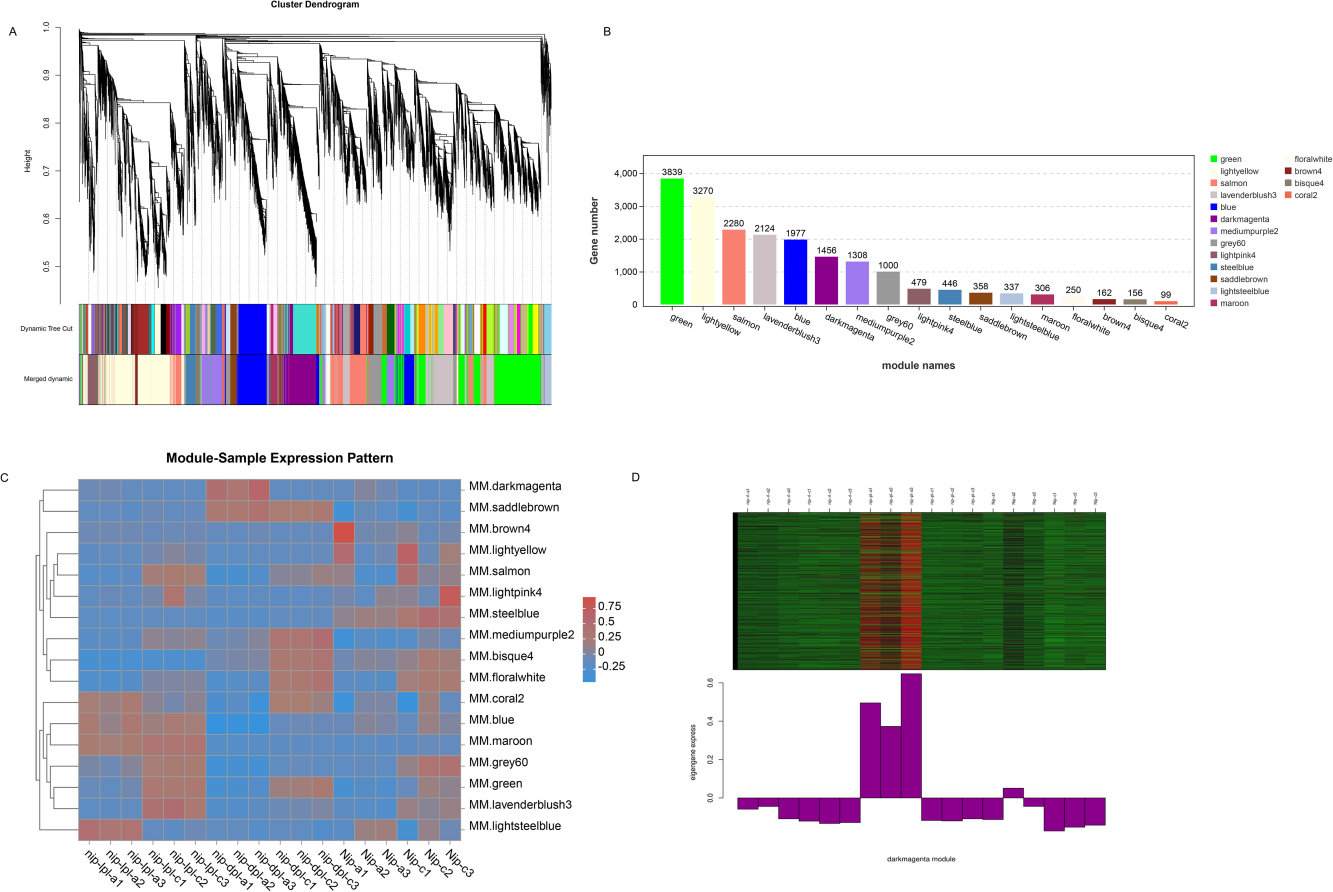
Weighted correlation network analysis modules of all DEGs established. **(A)** Tree graphs of DEGs by hierarchical clustering of topological overlapping dissimilarities. **(B)** The gene numbers in all modules. **(C)** Heat maps of the correlation between modules and samples. **(D)** Expression profiles of all co-expressed genes in the module. Darkmagenta color scale represents Z score. The bar graph shows the pattern of the corresponding co-expressed genes in the consistent expression module.

**Figure 9:**
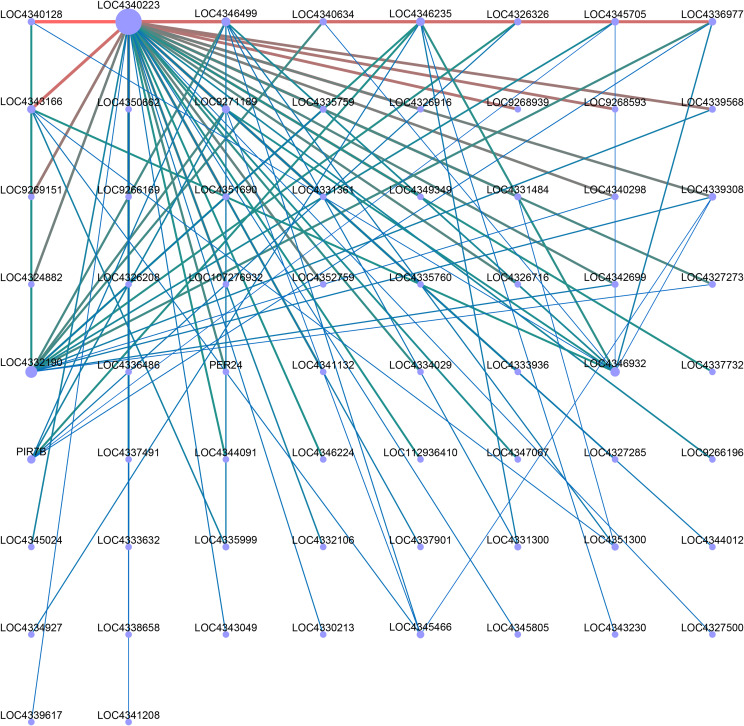
Co-expression regulatory network analysis of darkmagenta module. Dots in the network map represent gene names or gene ids, red lines represent positive regulation, and blue-green lines represent negative regulation.

In order to identify specific genes for color mutations in two different rice materials, we performed WGCNA analysis. We analyzed the correlation between the obtained modules, and a total of 17 modules were obtained with each containing 99 to 3839 genes (**Figures 8A, B**). In addition, the expression patterns of module genes in each sample were displayed by module eigenvalues, and the heat map of sample expression patterns was drawn (**Figure 8C**). Through the heat map of sample expression pattern, we found that the third leaf of *nip-dpl* with the highest anthocyanin accumulation in the darkmagenta module was significantly different from other leaves (**Figure 8D**), indicating that the genes in the darkmagenta module may be related to the anthocyanin accumulation process. The module contained 1456 genes related to genetic information processes (**Figure 8B**, **Supplementary Table S5**). In order to further study the molecular mechanism of anthocyanin accumulation, KEGG enrichment analysis was performed on the genes of the module. In KEGG analysis, genes were mainly enriched in flavonoid biosynthesis, phenylpropanoid biosynthesis, spliceosome, biosynthesis of secondary metabolites, metabolic pathway and other pathways (**Supplementary Figure S8**). Next, in the darkmagenta module, the DEGs that enriched in flavonoid biosynthesis and phenylpropanoid biosynthesis were used as central genes to construct a gene regulatory network (**Figure 9**), and to study the gene regulatory network of anthocyanin accumulation. Among them, the gene of *PIR7B* was found to be actively interacting with LOC4346499, LOC4346235, LOC4326326, LOC4345705, LOC4336977 and etc. The *PIR7B* b gene encodes an esterase that the protein accumulates in rice leaves (Urs et al., 1998).

### Gene expression validated by quantitative real-time polymerase chain reaction

**Figure 10:**
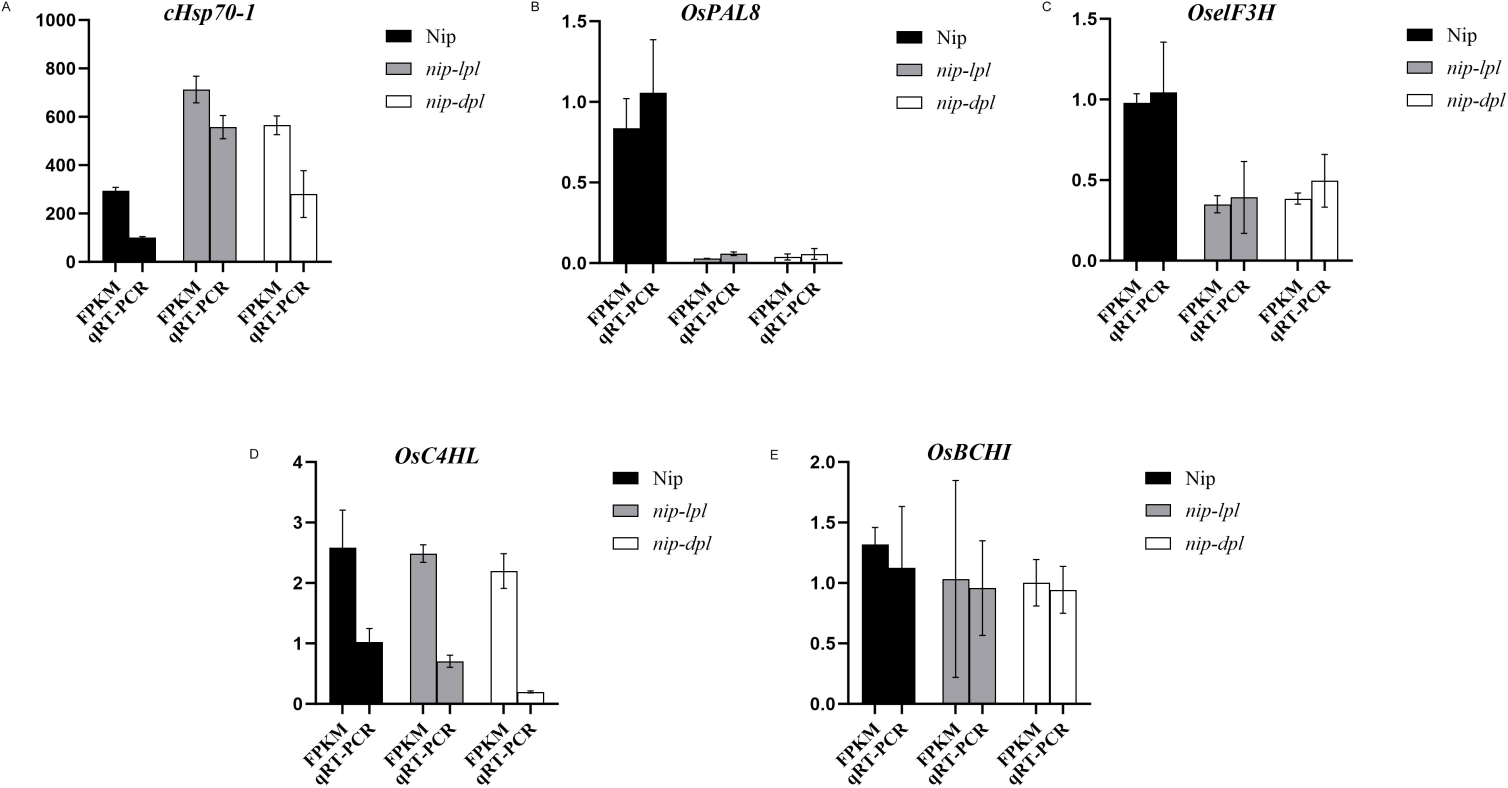
The expression profiles of qRT-PCR were compared with the results of RNA-Seq analysis. **(A)** *cHsp70-1*:stromal 70 kDa heat shock-related protein, chloroplastic-like, **(B)** *OsPAL8: Oryza sativa* L. phenylalanine ammonia-lyase, **(C)** *OseIF3H*: rice eukaryotic translation initiation factor 3 subunit h, **(D)** *OsC4HL*:cinnamate-4-hydroxylase (C4H) from a japonica type rice (*Oryza sativa* L. cv. *llpumbyeo*), **(E)** *OsBCHI*: synonym: *chlI*. The values on the vertical coordinate represent the gene expression levels of RNA sequencing and qRT-PCR.

To ensure the quality of high-throughput sequencing, several mRNAs were selected for qRT-PCR detection. The validated results were shown in **Figure 10**. In terms of the expression trend of *cHsp70-1, OsPAL8, OseIF3H, OsC4HL,OsBCHI*, qRT-PCR results were consistent with RNA-seq data.

### Subcellular localization analysis of selected genes

**Figure 11:**
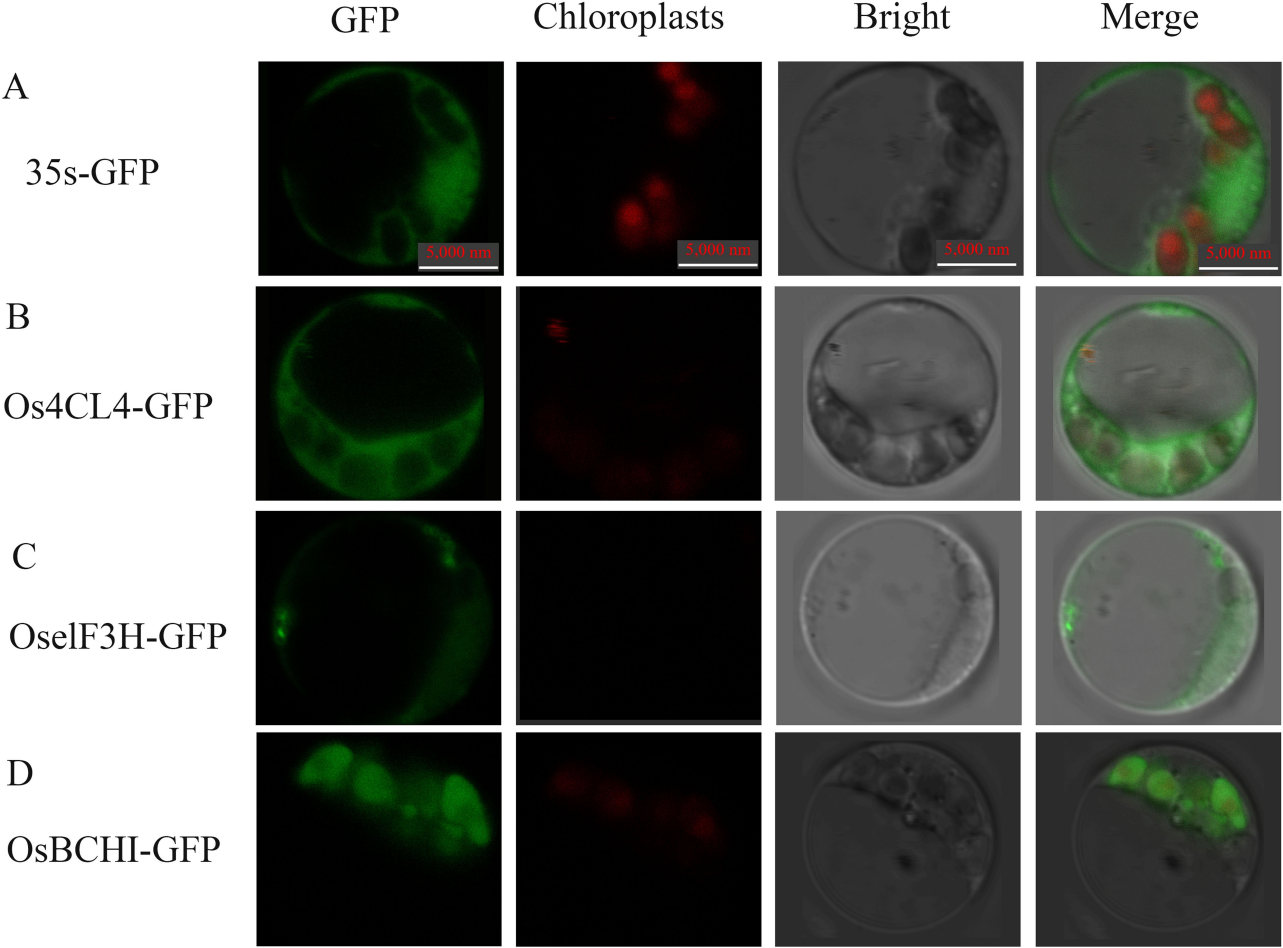
Confocal microscope observation of subcellular localization with green fluorescent protein (GFP) fusion organelle markers in rice protoplasts. Positive control **(A)**, Os4CL4 **(B)** is subcellular localized in the cytoplasm, OselF3H **(C)** is subcellular localized in the endoplasmic reticulum, and OsBCHI **(D)** is subcellular localized in the chloroplast. Take photographs under the 390-nm excitation light, Bright: Take photographs under the bright light. Scale bar = 5 µm.

The subcellular localization of the gene in rice protoplasts after fusion with green fluorescent protein (GFP) can clearly show the location of the target gene in the cell, which helps to further understand the possible mechanism of related genes regulating leaf color. The subcellular localization vectors of three DEGs, *Os4CL4*, *OselF3H* and *OsBCHI*, were constructed (**Figure 11**). These plasmids were transfected into rice protoplasts. Under confocal laser scanning microscopy, it was found that Os4CL4 protein was located in cytoplasm, OselF3H protein was located in endoplasmic reticulum, and OsBCHI protein was located in chloroplast. The results suggested that the protein OsBCHI may be involved in the synthesis of related pigments in chloroplasts, thereby affecting rice leaf color. These results also suggested that multiple cellular components of the cell may be synergistically involved in triggering the leaf color mechanism.

## Discussion

As a common mutation feature in plants, leaf color mutation is a vital source of germplasm resources for colorful leaf plants. Previous studies have shown that 208 leaf color mutants exist in rice (Deng et al., 2014). Rice can produce a variety of color variations in nature, including blue, reddish brown, light purple, deep purple and other color characteristics of leaves (Zhang P et al., 2021; Li et al., 2022b). These color variations are affected by many factors, such as the degree of pigment accumulation, light and gene regulation. The accumulation of pigments has a great impact on the change of leaf color. Some leaf color mutations have a red or purple phenotype, which is related to the synthesis and accumulation of anthocyanins (Wei et al., 2016). For example, two types of purple tea(‘Ziyan’ and ‘Zijuan’) are leaf color variations caused by excessive anthocyanin accumulation (Tan et al., 2023). Furthermore, researches showed that the content of anthocyanins in purple, red and blue leaf plants was much higher than that in other leaf color varieties (Mackon E et al., 2021; Sean et al., 2021; Xia et al., 2021). However, these results were lack of experimental basis and cannot well prove the mechanism of leaf color variation in rice. In order to find fundamental mechanisms for leaf color variation in rice, we used EMS to mutate the wild type rice Nip in the early stage, and constructed a rice mutant library, from which we obtained two kinds of rice with leaf color mutations, one of which was light purple in leaf color and panicle grain, the other was deep purple in leaf color and panicle grain. In the five-leaf stage, we took the RNA of two kinds of rice leaves and wild type leaves for transcriptome sequencing and found that the DEGs were involved in phenylalanine metabolism and terpenoid backbone biosynthesis pathway. This indicated that leaf color variation in rice may be closely related to these pathways. In addition, we explored the different agronomic traits of the three rice materials. It is worth noting that 1000-grain weight, number of grains per spik of *nip-lpl* and *nip-dpl* were much higher than wild-type Nip, the dry weight of *nip-lpl* was significantly higher than Nip, but the spikes per plant and 1000-grain weight of the two mutant plants was smaller than that of wild-type Nip. Dry matter accumulation and 1000-grain weight of rice have many influences on rice quality, which together determine the yield and quality characteristics of rice (Lin et al., 2003). Dry matter is the sum of photosynthetic products accumulated in rice, which directly affects the grain fullness and nutrient composition. The 1000-grain weight reflects the fullness of a single grain and is a cooperative index of yield and quality, high 1000-grain weight may be accompanied by increased chalkiness, affecting the appearance of rice. High-quality rice varieties usually have a low 1000-grain weight, but reasonable distribution of dry matter, less chalkiness, and good taste (Ichsan, 2021). Therefore, we speculated that the yield and quality of the two mutant rice *nip-lpl* and *nip-dpl* were higher than that of the wild rice Nip.

It is well known that anthocyanin is a flavonoid polyphenol compound. Firstly, phenylalanine, the precursor of anthocyanin synthesis, is catalyzed by PAL, C4H and 4CL to form the primary substrate 4CoA, which is the initial stage of flavonoid metabolism (Mizuno T et al., 2021; Li et al., 2022a). Meanwhile, UV-B upregulates the genes that regulate C4H (cinnamate 4-hydroxylase). Secondly, 4CoA and malonyl-CoA are catalyzed by CHS to form chalcone, which is then isomerized to trihydroxy flavanone under the action of CHI and then hydroxylated by F3H to form dihydroflavonol. Dihydroflavonols are catalyzed by DFR and ANS to form colored anthocyanins (Yuan et al., 2009; Liao J, 2015; Dai Y, 2022; Sharma H and Dhatt, 2022; Li X et al., 2023). The KEGG enrichment analysis of differentially expressed genes revealed significant enrichment in the phenylalanine biosynthesis pathway and the flavonoid biosynthesis pathway (**Figure 6**). This compound can catalyze the accumulation of dihydroxyflavone while also generating gibberellic acid (Mackon E et al., 2021; Xia et al., 2021). In addition, compared with wild-type Nip, the genes related to phenylpropionic acid and flavonoid synthesis in the rice materials *nip-lpl* and *nip-dpl* were up-regulated to varying degrees, including *PAL*, *4CL*, *C4H*, *CHS*, *CHI* and *F3H*. This has been confirmed by qRT-PCR (**Figure 10**). These results suggested that increased levels of these enzymes may lead to increased anthocyanin content in the mutants. We speculated that increased levels of these enzymes lead to significant accumulation of anthocyanins.

The synthesis and accumulation of anthocyanin is a diversified process, which is not only affected by the expression of genes in plants, but also by the external environment (Zhou, 2021; Tapia G and GaeteEastman, 2022). Light is one of the most important external conditions. Studies have shown that high-intensity UV-B irradiation of apples will increase the activity of enzymes that promote anthocyanin synthesis in the epidermis, which is conducive to the synthesis and accumulation of anthocyanins (Chen et al., 2019). Therefore, it is speculated that the leaf color mutations of the two mutants *nip-lpl* and *nip-dpl* were also subjected to long-term high intensity light irradiation which the experimental materials planted in the month with the strongest ultraviolet rays in a year, thereby increasing the activity of C4H enzyme in leaves, resulting in the accumulation of anthocyanins.

Rice ribosomes are located in mesophyll cells and participate in intracellular protein synthesis. Meanwhile, the ribosomal pathway was significantly enriched in the KEGG analysis of rice materials *nip-lpl* and *nip-dpl* (**Figure 6**). According to the above results, we speculated that the synthesis frequency of anthocyanin-related proteins in the mutant was accelerated in ribosomes, leading to the accumulation of anthocyanin content. In addition, in the KEGG analysis of the two mutant materials, the genes of β-glucosidase and peroxidase were up-regulated in the phenylpropionic acid biosynthesis pathway. β-glucosidase can hydrolyze cellobiose and cellooligosaccharides in plants to produce glucose, while peroxidase can scavenge reactive oxygen species in plants (Suhartatik et al., 2019; Sirilun et al., 2022; Ma J and Liu, 2023). Therefore, we speculated that these pathways and gene up-regulation lead to increased glucose content and enhanced photosynthesis in mutant rice leaves, thus promoting anthocyanin synthesis.

In this study, we selected two rice mutant varieties, light purple rice *nip-lpl* and deep purple rice *nip-dpl*. By transcriptome analysis of them and wild-type rice Nip, 71 common DEGs were identified among the three rice materials, and 1104, 2288, 2383 common DEGs were identified in Nip vs *nip-lpl*, Nip vs *nip-dpl*, *nip-lpl* vs *nip-dpl*. GO analysis showed that DEGs were highly enriched in terms of metabolic processes, catalytic activity, and cells. KEGG analysis showed that these DEGs had different degrees of enrichment in terpenoid backbone biosynthesis pathway and phenylpropionic acid biosynthesis pathway. The abundant expression pathways in different leaf positions of the three rice materials were also similar to the above pathways (**Figure 6**). This suggested that these pathways may be the key pathways leading to anthocyanin accumulation in rice leaves.

Over the past decade, extensive research on model plants and various fruits has uncovered anthocyanin metabolism pathways and biosynthetic genes, demonstrating a certain level of conservation (Jaakola, 2013). Anthocyanin biosynthesis is governed by upriver structural genes (*CHS*, *CHI*, and *F3H*) and specific biosynthesis genes (*F3’H*, *F3’5’H*, *DFR*, *ANS* and *UFGT*). A comprehensive analysis of the rice genome and transcriptome has identified several candidate hub genes involved in anthocyanin synthesis (Huang et al., 2024). Notably, the hub genes *PAL*, *CHS* and *CHI* showed elevated expression in *nip-lpl* and *nip-dpl* (**Figure 7**), leading to increased anthocyanin content in *nip-lpl* and *nip-dpl* samples relative to Nip. The study identified three *PAL* genes—ncbi_4330034, ncbi_4330036 and ncbi_4336415, one *CHS* gene- ncbi_4350636 and one *CHI* gene-ncbi_4334588 that exhibited higher expression levels in *nip-lpl* and *nip-dpl* compared to Nip. The differentially expressed levels of these candidate genes across the three rice cultivars offer significant insights into their role in rice leaf color and anthocyanin accumulation.

However, this study has potential limitations. Firstly, the three rice cultivars were collected over a year. Environmental changes affect the leaf color of plants. Due to climate change, there may be differences in the anthocyanin accumulation of rice leaf collected in different years. To draw a clear conclusion, a long-term investigation is necessary. Secondly, although we investigated the anthocyanin accumulation of three rice cultivars of Nip, *nip-lpl* and *nip-dpl*, the number of rice mutants was relatively small. Future research should use more rice mutants to validate the results of this study.

## Conclusion

In summary, analyses of the phenotypic and transcriptome analysis were conducted on the japonica rice cultivar nipponbare (Nip) and its two purple leaf mutants, designated *nip-light purple leaf (nip-lpl)* and *nip-deep purple leaf (nip-dpl)*. KEGG analysis showed that the DEGs were significantly enriched in phenylalanine biosynthesis, terpenoid backbone biosynthesis, secondary metabolite biosynthesis, and hormones. Additionally, WGCNA showed that the darkmagenta module was associated with the purple color mainly due to the accumulation of anthocyanin in the leaves of the mutant rice. This module revealed three pathways for anthocyanin synthesis: phenylalanine could be catalyzed by phenylalanine lyase and cinnamic acid hydroxylase, etc., to generate dihydroxyflavone and ultimately anthocyanin. Furthermore, the elevated expression of three kinds of hub genes (*PAL*, *CHI* and *CHS*) in *nip-lpl/dpl* leads to increased anthocyanin content relative to Nip. Our findings offer an in-depth insight into the molecular mechanisms triggering leaf purple color in the rice mutant *nip-lpl/dpl* but also will contribute greatly to identified potential genetic engineering targets for breeding anthocyanin-rich rice. The present findings provide valuable information for anthocyanin metabolism and potential candidate genes for further genetic manipulation of anthocyanin biosynthesis in rice.

## Data Availability

The data presented in the study are deposited in the OMIX repository, accession number OMIX010126-01.
