# Supplementary material for: Genome-wide transcriptome analysis reveal the molecular mechanism for triggering the formation of purple leaves in rice mutants nip-lpl and nip-dpl
Source: Front Plant Sci. 2025 May 30;16:1584423. doi: 10.3389/fpls.2025.1584423 (PMC12162559; doi:10.3389/fpls.2025.1584423)
Supplement: Supplementary file 1 [file DataSheet1.docx]

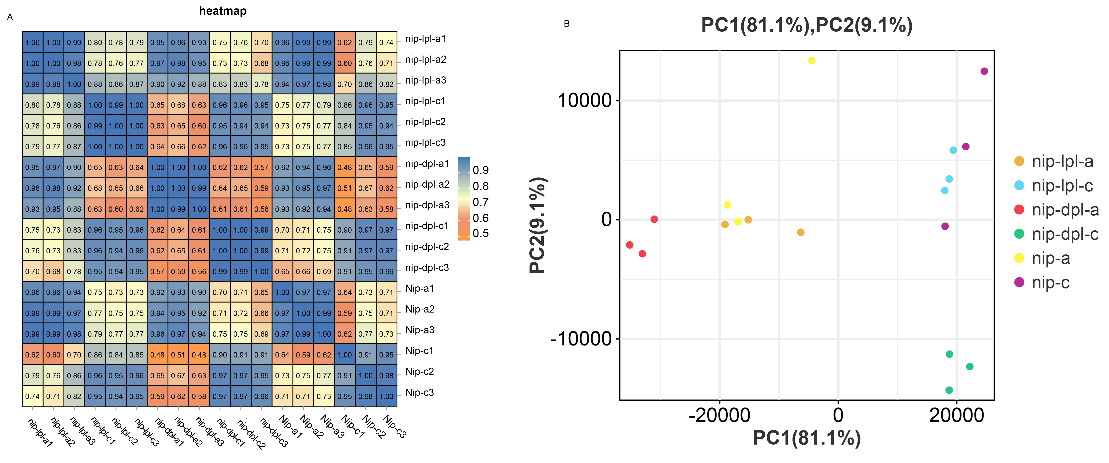


**Figure S1.** Correlation analysis among the samples including nip-a, nip-c, *nip-lpl-a*, *nip-lpl-c*, *nip-dpl-a* and *nip-dpl-c*. (A) The correlation coefficient between each pair of samples showing the biological repeatability. The color intensity represents the p-value. (B) Principal component analysis (PCA) showing the correlation among samples at the level of two PCs.


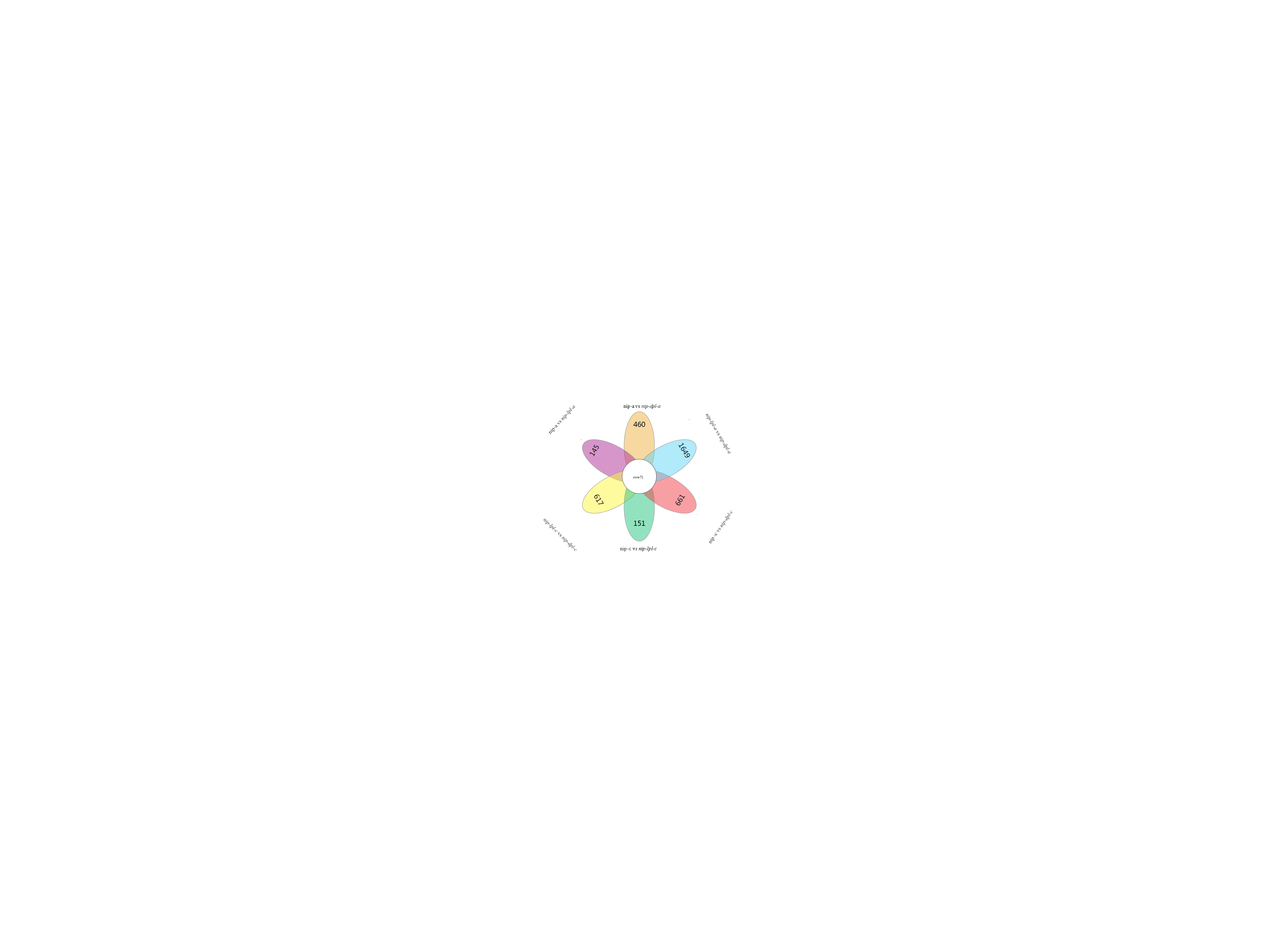


**Figure S2**: Common DEG analysis in comparison of three different rice materials.


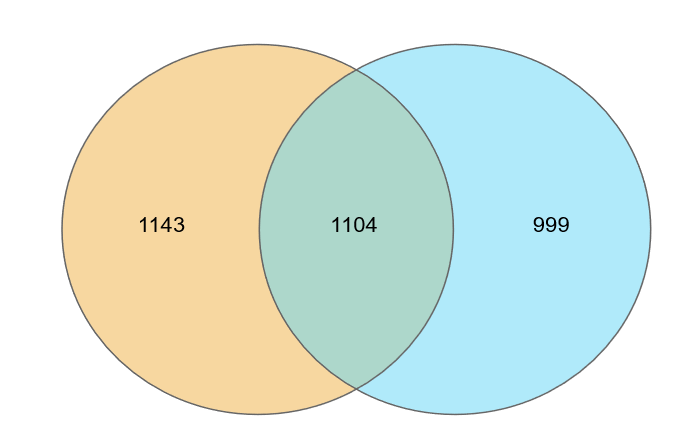


A

*nip-a vs nip-lpl-a nip-c vs nip-lpl-c*

B


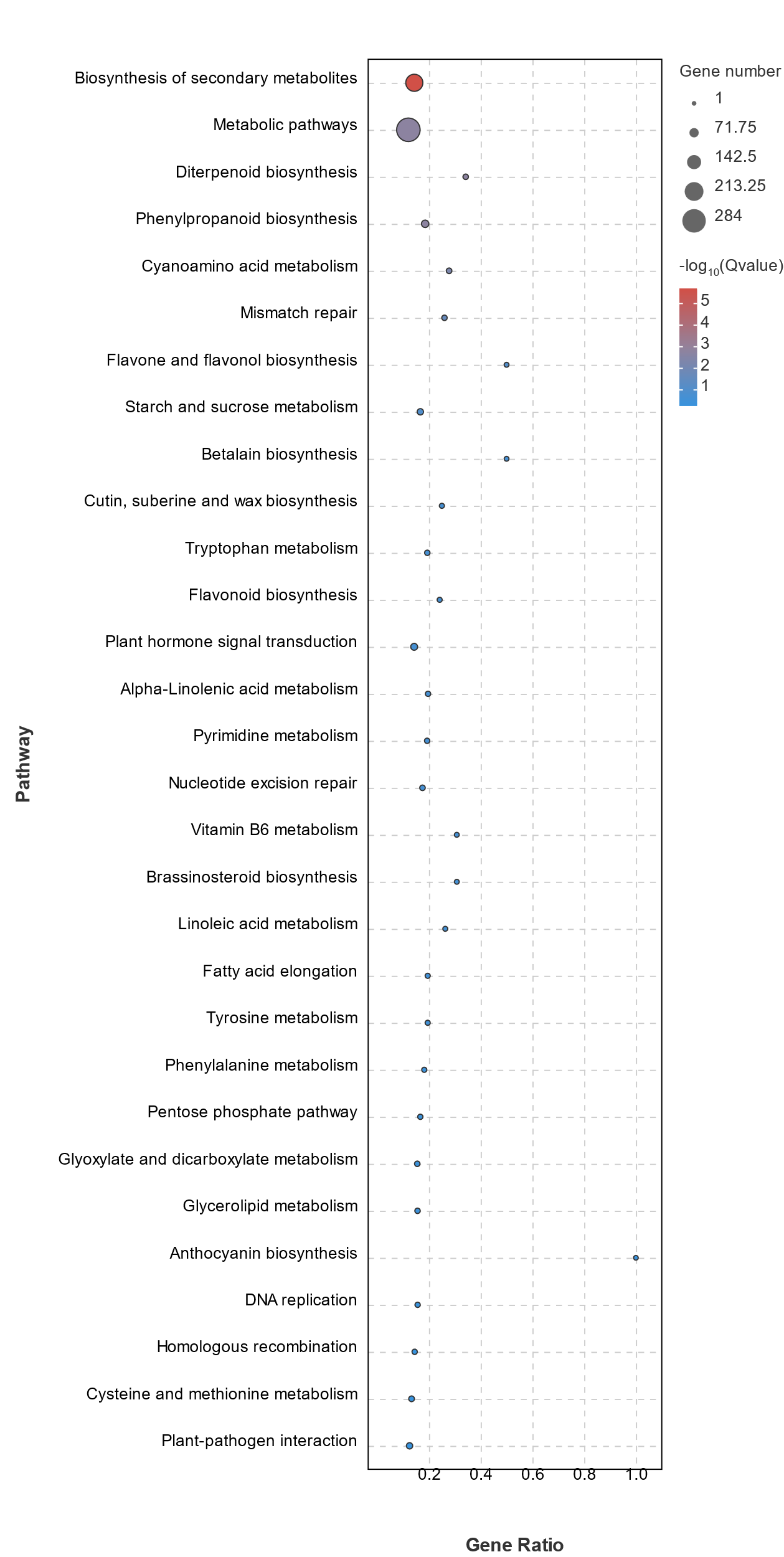


**Figure S3**: Common DEG analysis of two different rice materials. (A) Venn diagram for comparison of the number of wild type and mutant DEG. (B) The top 30 are rich in KEGG pathway.


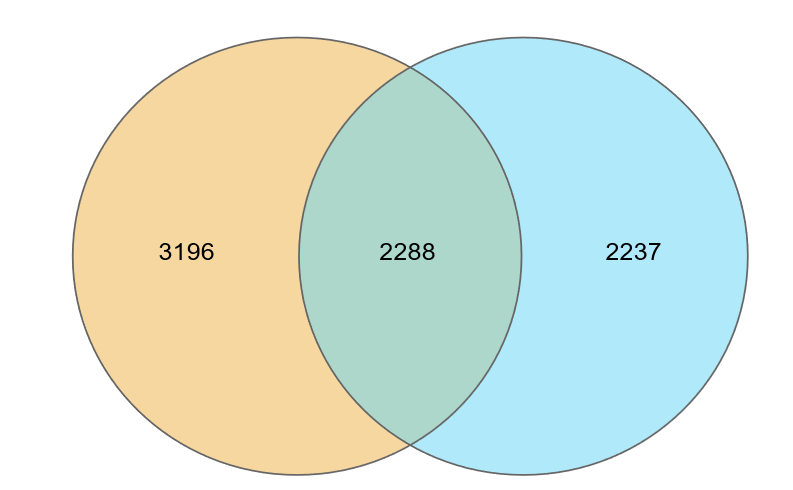


A

*nip-a vs nip-dpl-a nip-c vs nip-dpl-c*


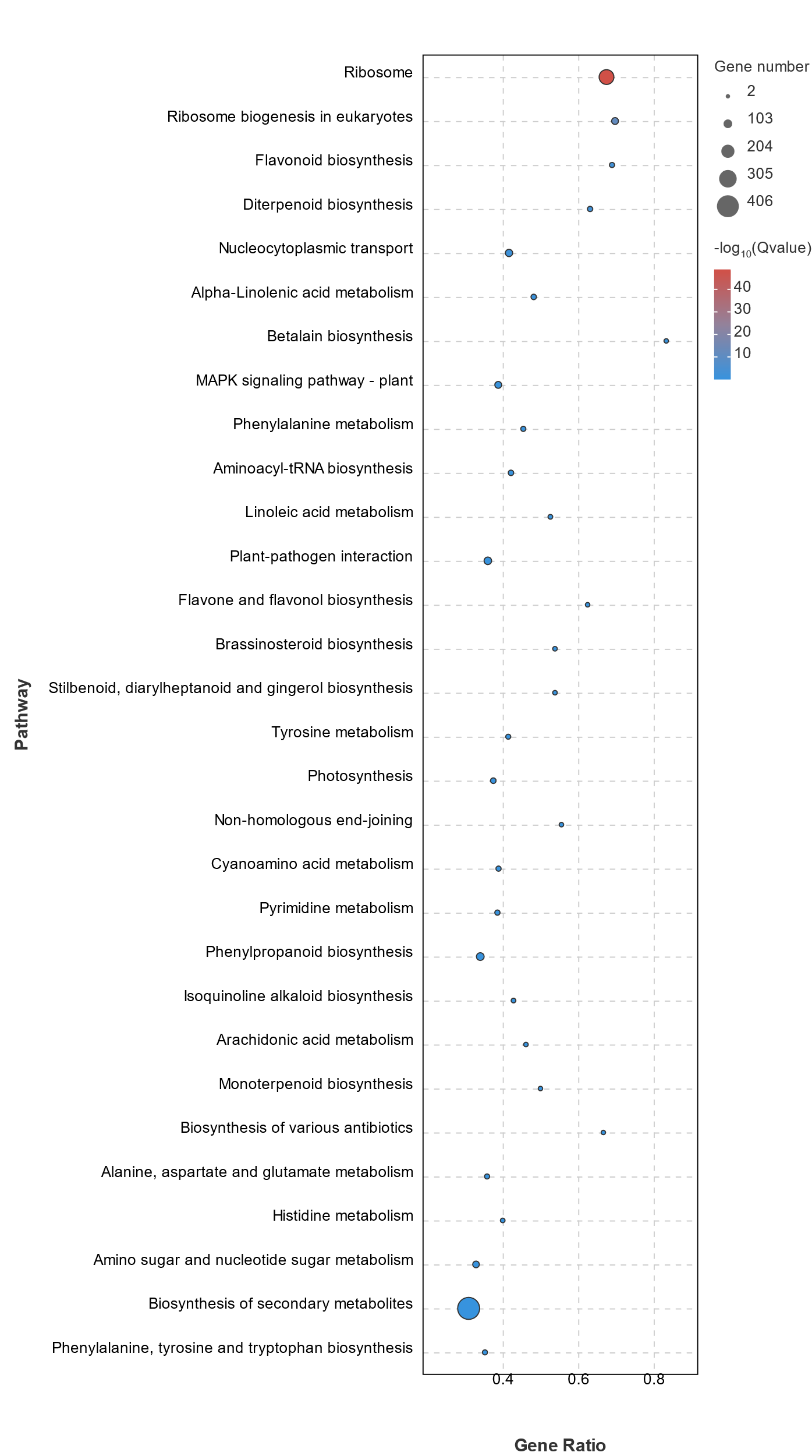


B

**Figure S4**: Common DEG analysis of two different rice materials. (A) Venn diagram for comparison of the number of wild type and mutant DEG. (B) The top 30 are rich in KEGG pathway.

*nip-lpl-a* vs *nip-dpl-a nip-lpl-c* vs *nip-dpl-c*


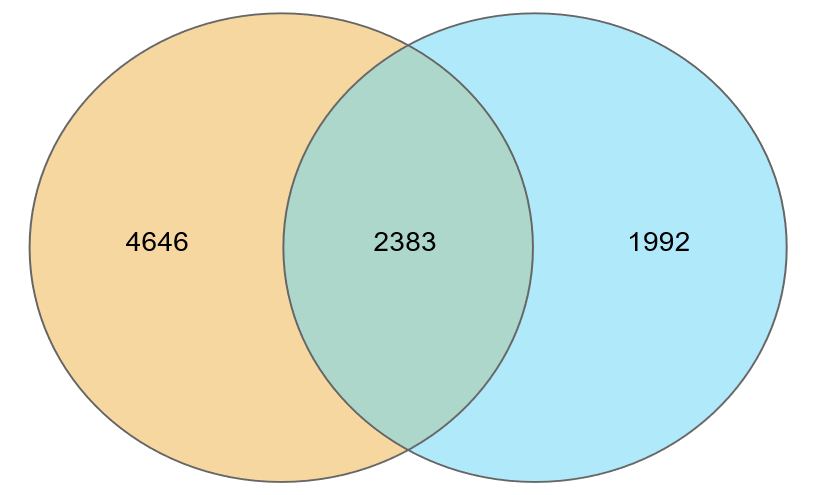


A

*nip-lpl-a* vs *nip-dpl-a nip-lpl-c* vs *nip-dpl-c*

B


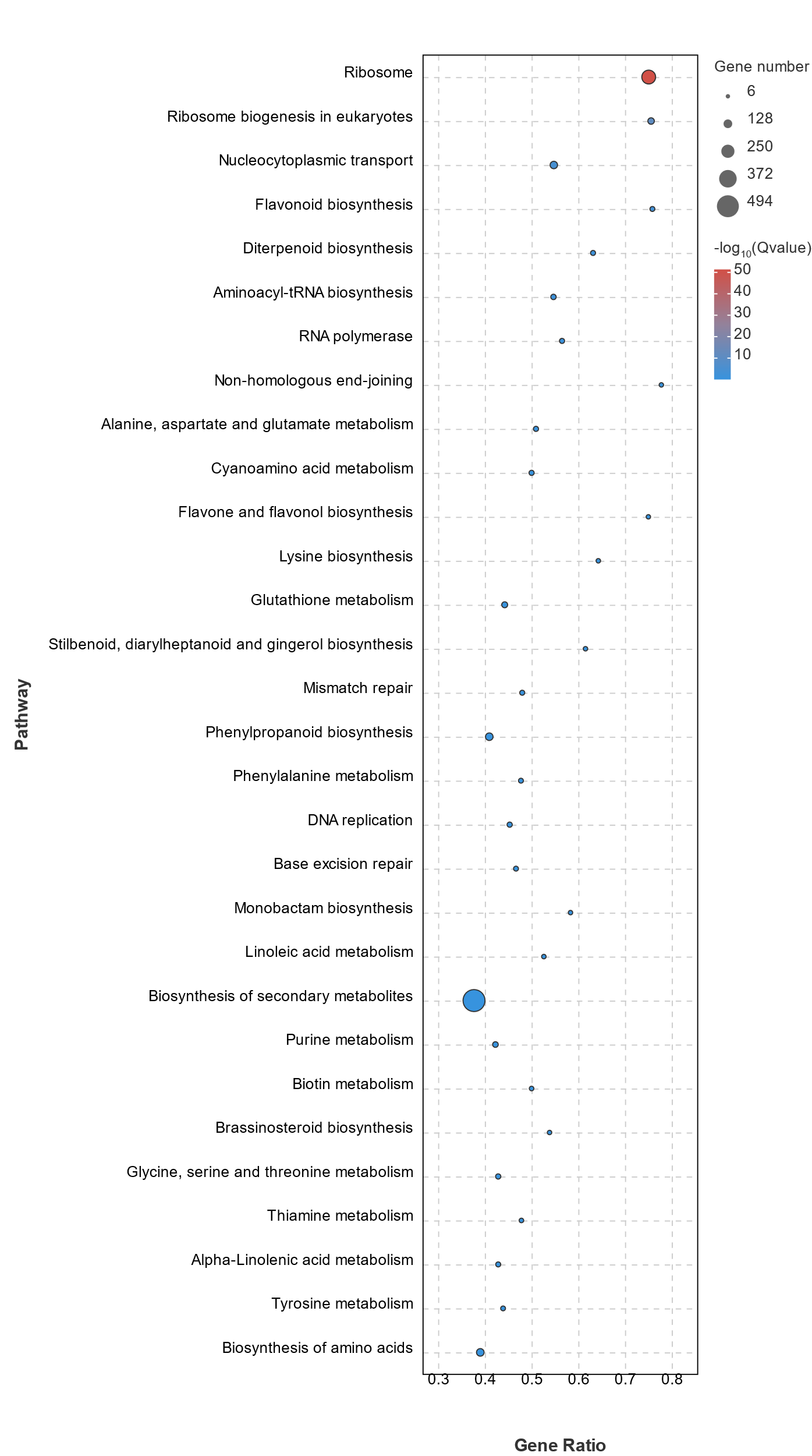


**Figure S5**: Common DEG analysis of two different rice materials; (A) Venn diagram for comparing the number of DEGs of *nip-lpl* and *nip-dpl*; (B) The top 30 are rich in KEGG pathway.


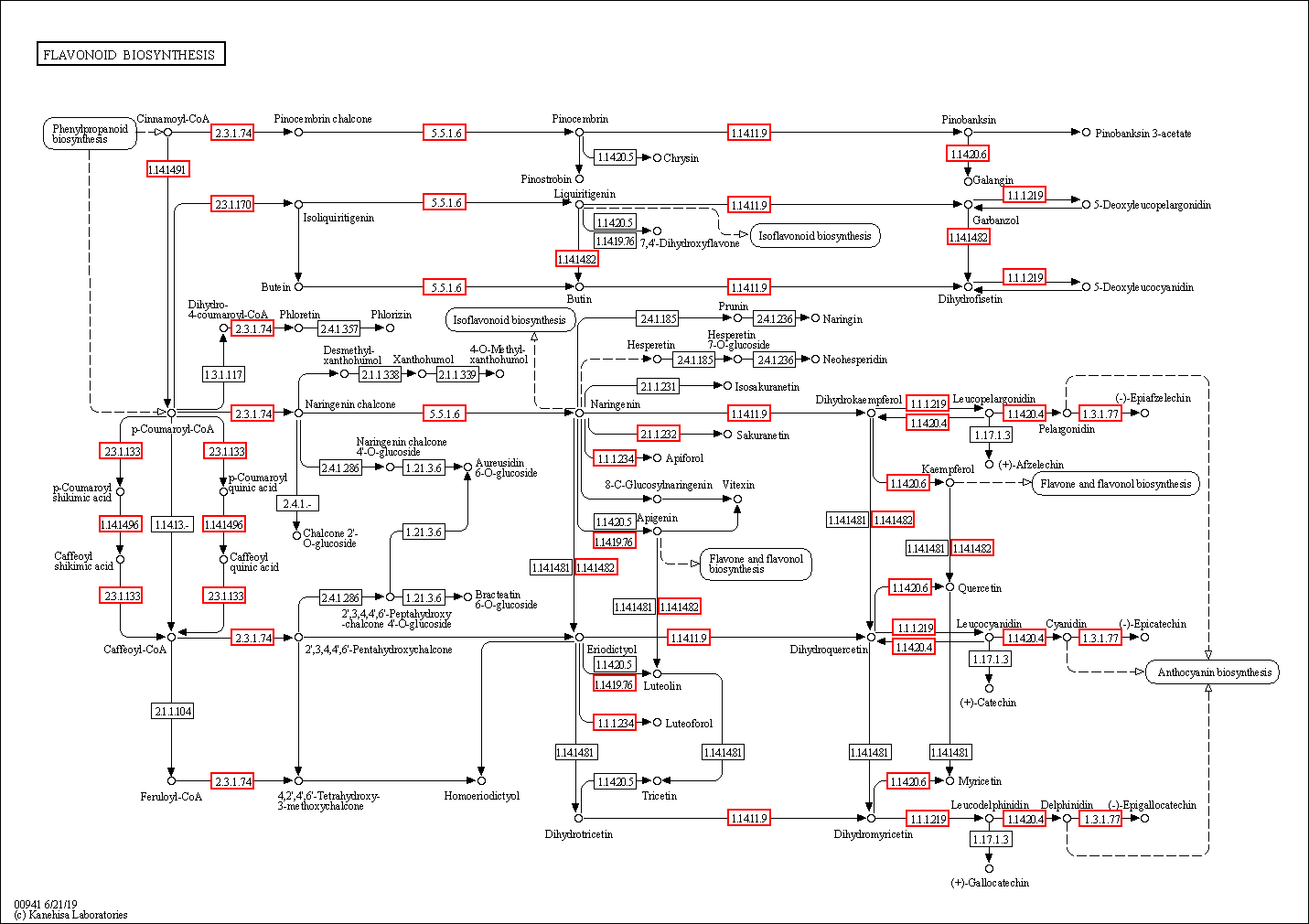

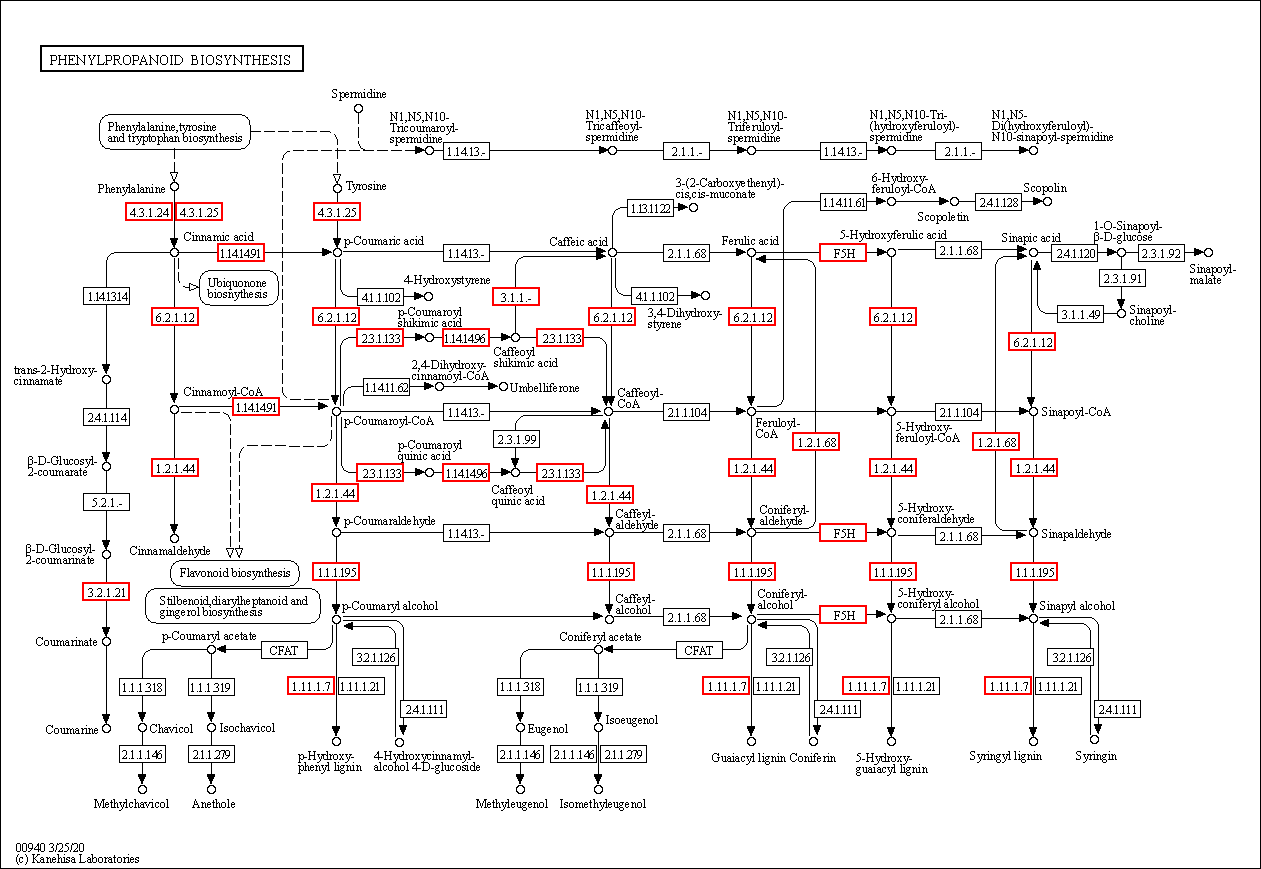


A

B


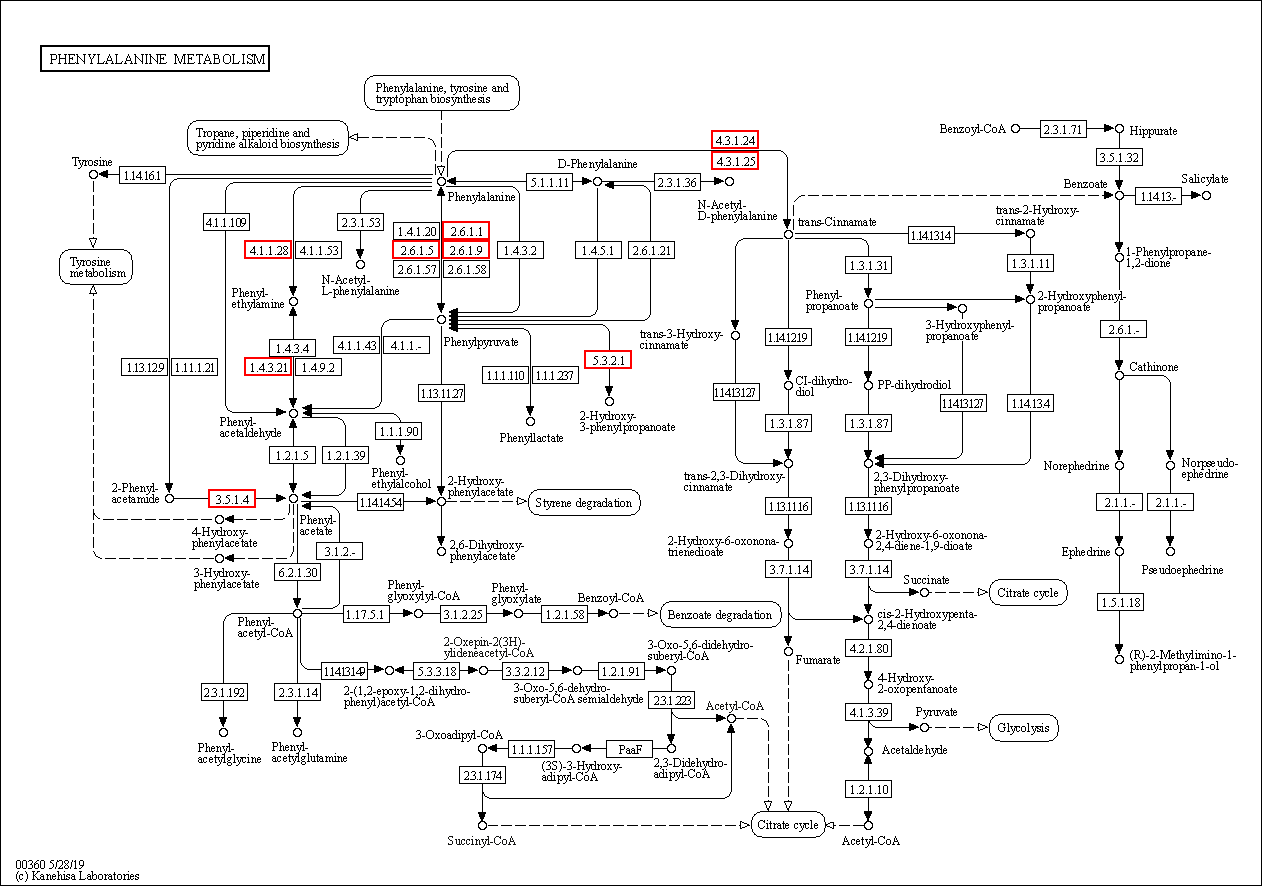


C

**Figure S6**: Enrichment pathway of KEGG gene in Ni**p** vs *nip-dpl*. (A) Flavonoid biosynthesis; (B) Phenylpropanoid biosynthesis;(C) Phenylalanine metabolism. KEGG path database is from Kanehisa laboratory.


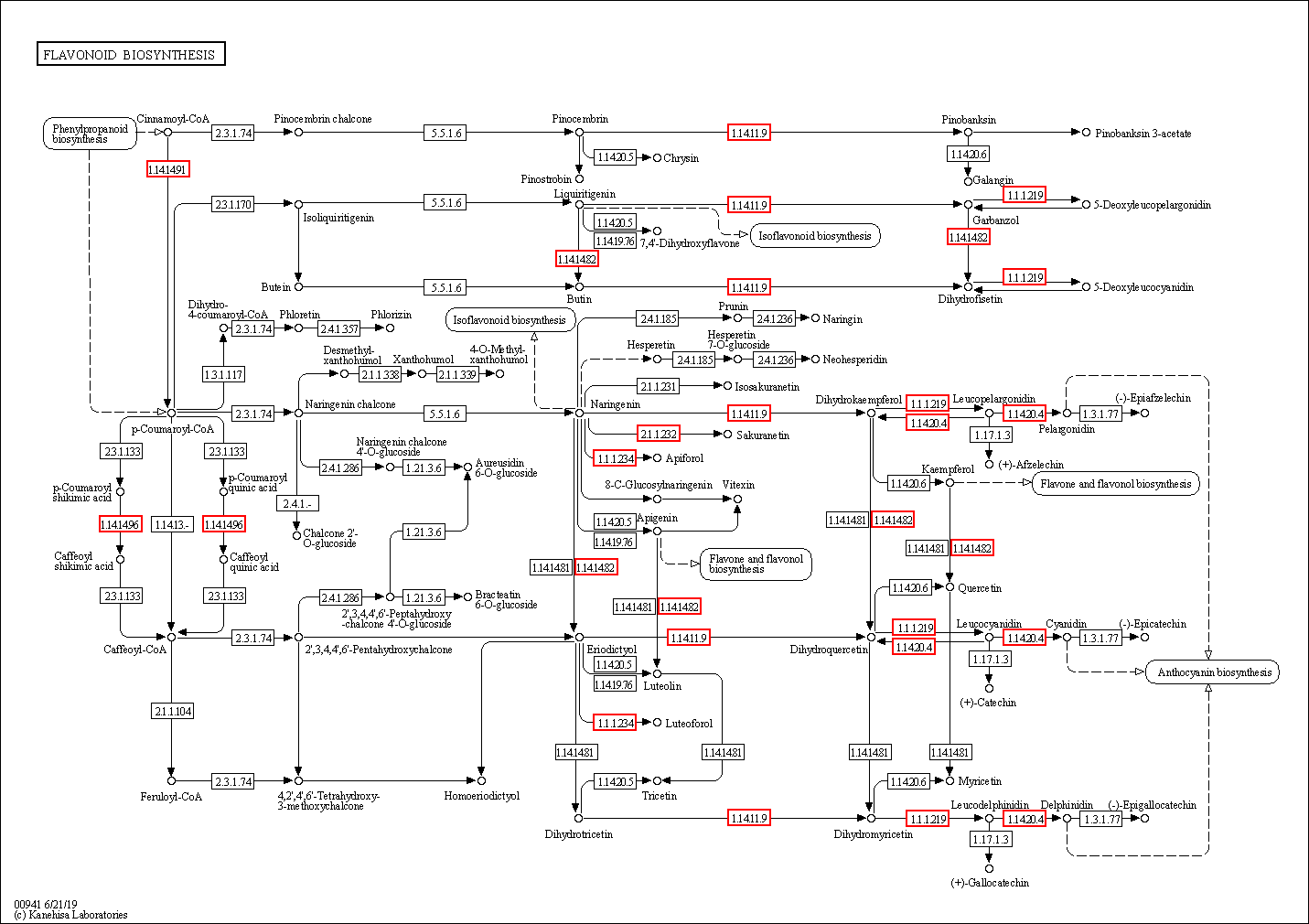


A


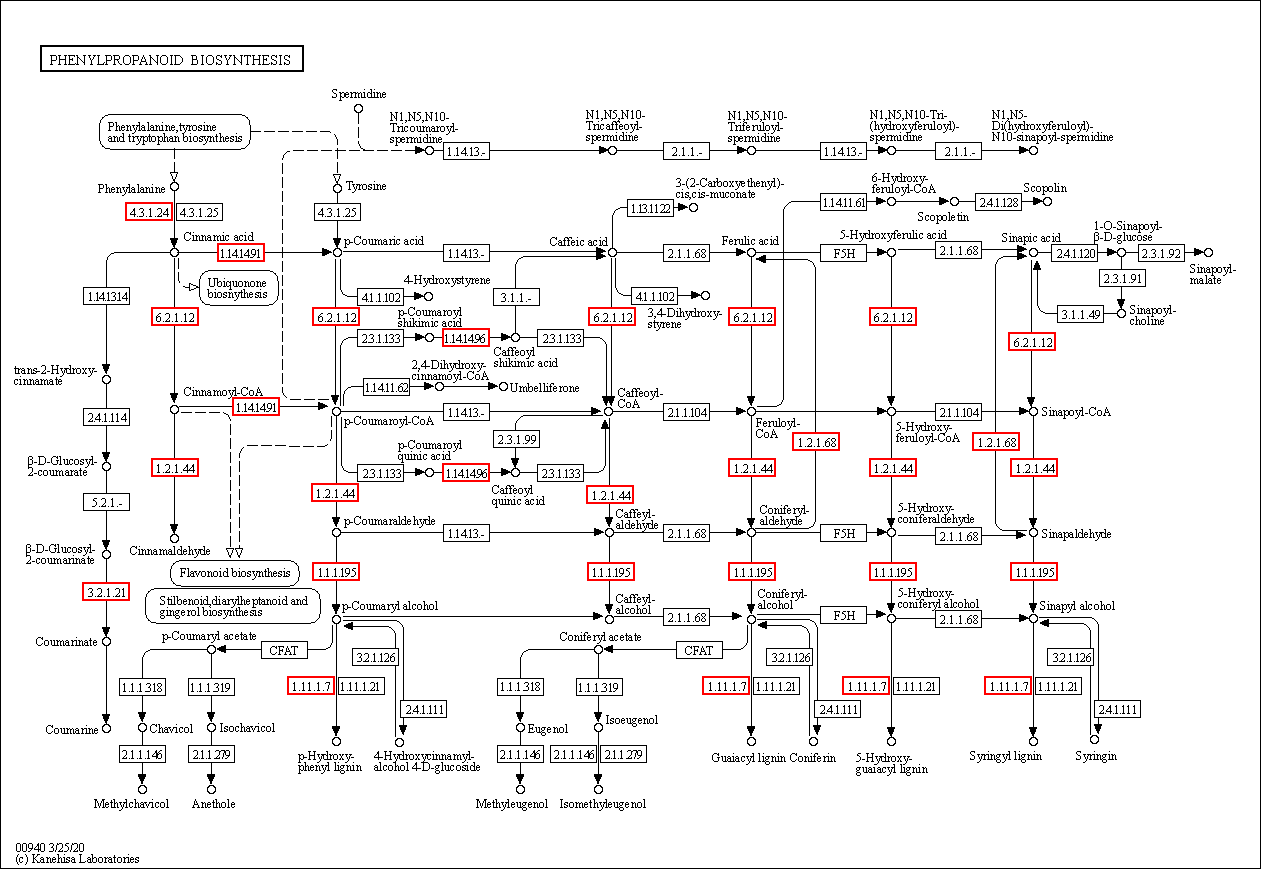


B


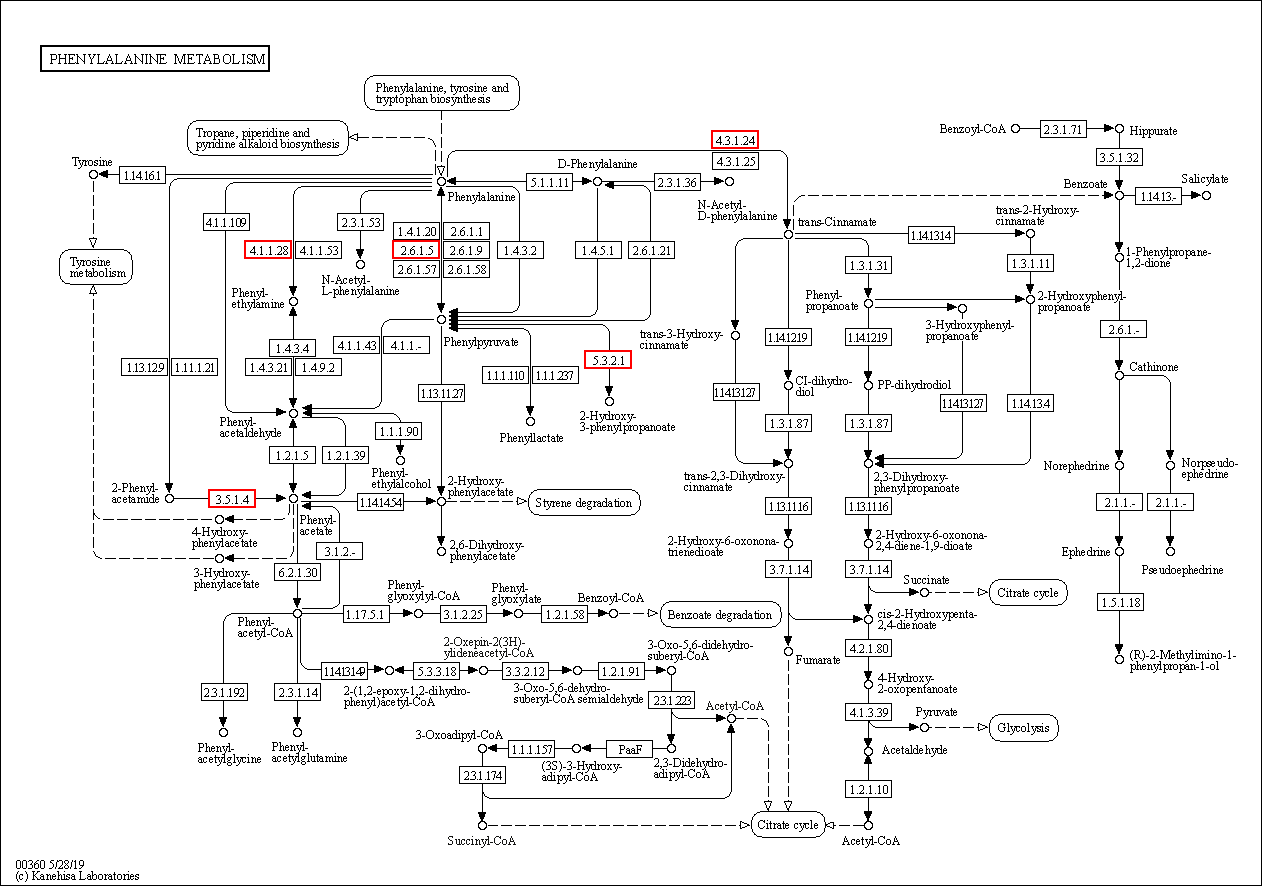


C

**Figure S7**: Enrichment pathway of KEGG gene in Nip vs *nip-lpl*. (A) Flavonoid biosynthesis; (B) Phenylpropanoid biosynthesis; (C) Phenylalanine metabolism. KEGG path database is from Kanehisa laboratory.


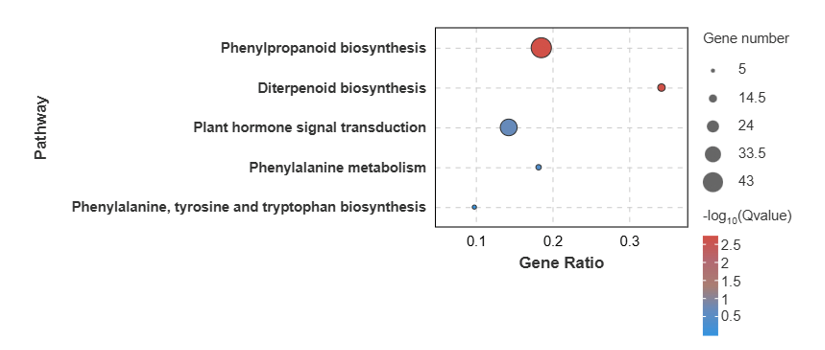


**Figure S8** KEGG pathway enrichment analysis module darkmagenta gene.
