# Supplementary material for: Genome-wide transcriptome analysis reveal the molecular mechanism for triggering the formation of purple leaves in rice mutants nip-lpl and nip-dpl
Source: Front Plant Sci. 2025 May 30;16:1584423. doi: 10.3389/fpls.2025.1584423 (PMC12162559; doi:10.3389/fpls.2025.1584423)
Supplement: Supplementary file 2 [file Table1.docx]

| F3H-F | GCGAGGAGTGGGGCATCTTC |
| --- | --- |
| F3H-R | CCGGACATGTCGAACCGGAG |
| 4CL4-F | TCCGCATGGTTATGTCCGGC |
| 4CL4-R | CCCCGGATGCAAATCTCCCC |
| CHSP70-F | AGCCTACCTCGGCTCCAACA |
| CHSP70-R | GTGTCACCAGCAGTGGCCTT |
| PAL8-F | GCGTCAACTCTCTCGGCCTC |
| PAL8-R | CTCTCCTCGAGTTGGCGCAG |
| C4HL-F | ACCTCAACCACCGCAACCTG |
| C4HL-R | ACCTTGCCGGTGAAGAACGG |
| CH1-F | CGACGTCTTCGCCATCACCA |
| CH1-R | CCCACCTCCTCCAGCTCCTT |

Table S1 The primers of genes for RT-qPCR
